# Comprehensive characterisation of the active ingredients of *Smilax glabra* Roxb based on chemical fingerprinting, metabolic fingerprinting and pharmacodynamic fingerprinting

**DOI:** 10.3389/fphar.2025.1519054

**Published:** 2025-04-23

**Authors:** Wenqing Shi, Mengqi Jia, Xiao Li, Xin Zhao, Chenxi Wang, Guorong Fan, Yuefen Lou

**Affiliations:** ^1^ Department of Pharmacy, Shanghai Fourth People’s Hospital, School of Medicine, Tongji University, Shanghai, China; ^2^ Department of Clinical Pharmacy, Shanghai General Hospital, Shanghai Jiaotong University School of Medicine, Shanghai, China; ^3^ School of Medicine, Shanghai University, Shanghai, China; ^4^ Department of Clinical Pharmacy, Shanghai Jiao Tong University School of Medicine, Shanghai, China; ^5^ School of Pharmacy, Shanghai Jiao Tong University, Shanghai, China

**Keywords:** *Smilax glabra* Roxb, flavonoids, metabolism, tissue distribution, hyperuricemia

## Abstract

**Background:**

Smilax glabra Roxb (SGR) is a traditional Chinese medicine known for its medicinal and edible properties, with a long history of clinical use in treating hyperuricemia (HUA). However, current research has primarily focused on ethanol extracts, leaving the active ingredients and mechanisms responsible for the uric acid-lowering effects of SGR standard decoction unclear.

**Methods:**

Firstly, the chemical components in the standard decoction of SGR were characterized by ultra-performance liquid chromatography-quadrupole time-of-flight mass spectrometry (UPLC-Q-TOF/MS), and the pharmacodynamic experiments in mice with a high uric acid model were used to rapidly screen out the uric acid-lowering active ingredient group. Secondly, metabolic fingerprinting and tissue distribution analysis were performed on plasma and tissue samples from rats orally administered with SGR, respectively, to identify the key components and target organs. Finally, the core targets of these active ingredients were screened and analyzed by molecular docking technology.

**Results:**

We fractionated the ingredients of the SGR standard decoction into large and medium polar compound groups using macroporous resin, identifying 20 components. Then, through the pharmacodynamic experiment in hyperuricemic mice, we verified that the group of medium polar compounds in SGR had significant uric acid-lowering effects. In the metabolic fingerprinting analysis, 8 flavonoids and 24 metabolites were screened in the plasma of SD rats. Tissue distribution analysis revealed that the liver, intestine, kidney, and stomach were the main target organs for the active ingredients, with neoastiblin, astilbin, neoisoastiblin, isoastiblin, engeletin, and metabolites M01, M08, and M15 being the most widely distributed. Molecular docking confirmed that metabolites M08, M11, M15, and M16 exhibited strong binding activities with the target proteins CNT2, XOD, and URAT1.

**Conclusion:**

This study provides valuable references and insights into the pharmacodynamic substance basis and mechanism of action of SGR standard decoction for HUA treatment, through comprehensive analyses of chemical, metabolic, and pharmacodynamic fingerprints.

## 1 Introduction

Hyperuricemia (HUA) has recently gained increasing attention and is now the fourth most common basal metabolic disorder, following hypertension, hyperlipidemia, and hyperglycemia. HUA is caused by purine metabolism disorders or inadequate uric acid excretion (Gliozzi et al., 2016; Major et al., 2018). Elevated UA levels are closely linked to an imbalance in UA production and excretion processes. Concentration type nucleoside transporter 2 (CNT2) is the primary purine nucleoside transporter involved in intestinal absorption and transfer. Inhibiting CNT2 to reduce purine absorption in the gastrointestinal tract may provide an effective means of preventing HUA (Tatani et al., 2016). Xanthine oxidase (XOD) is responsible for the final two steps in the purine metabolic pathway and is the key enzyme that catalyzes the conversion of hypoxanthine and xanthine to UA (Lin et al., 2019). Additionally, the kidney filters UA through the glomeruli, reabsorbing approximately 90% of it into the bloodstream via the apical urate transporter 1 (URAT1) (Cho et al., 2015). Therefore, the target proteins CNT2, XOD, and URAT1 represent key therapeutic targets for inhibiting uric acid synthesis, transport, and absorption. Currently, there are two main types of drugs used in the treatment of hyperuricemia. One is mainly used to inhibit the uric acid precursor enzyme (xanthine oxidase), such as allopurinol and febuxostat, which can reduce serum uric acid levels by reducing purine synthesis. But allopurinol has adverse effects such as allergic syndrome and eosinophilia (Sharbaf et al., 2024), while febuxostat can cause skin rashes and heart failure (Sugawara et al., 2024). Another, mainly by promoting uric acid excretion, such as benzbromarone, but it causes liver and kidney damage (Kang et al., 2021). Therefore, it is urgent to find a method that is effective in its action and safer.

*Smilax glabra* Roxb (SGR), commonly known as “Tu fuling” in Chinese, is a rhizome of the Liliaceae plant that has been widely used as a functional food and herbal medicine throughout Southeast Asia, particularly in China, India, Vietnam, and Thailand (Wu et al., 2022a). SGR is known for its efficacy in addressing various conditions through its use in functional foods and folk remedies, particularly for dampness-heat, detoxification, and inflammation. Pharmacological studies have identified that the active components of SGR primarily include flavonoids, phenolics and phenolic acids, stilbenes, organic acids, and phenylpropanoids, among others (Hua et al., 2018). Flavonoids, a class of polyphenolic molecules with a benzo-γ-pyrone structure, are well-recognized (Rudrapal et al., 2021) for their broad range of pharmacological activities, including antioxidant (Yang et al., 2017), anti-inflammatory (Wang et al., 2019), antibacteria (Xu et al., 2013), hepatoprotective (Wang et al., 2021), and anti-hyperuricemic effects (Xue et al., 2023). These effects are structure-dependent, with the chemical properties of flavonoids being influenced by their structural class, degree of hydroxylation, substitutions, conjugations, and polymerization (Kumar and Pandey, 2013). Flavonoids such as astilbin, isoastilbin, neoastilbin, neoisoastilbin, engeletin, isoengeletin, and quercitrin are not only used as markers for quality control in preparing SGR in the Chinese Pharmacopoeia (e.g., astilbin), but are also widely reported for their medicinal properties (Dai et al., 2015).

Nowdays, studies related to SGR include chemical and pharmacodynamic effects of ethanol extracts, but the extraction methods are outdated and prone to waste of organic reagents and are not applicable for clinical use. We tried to find an extraction method with high extraction efficiency and more suitable for clinical use. SGR standard decoction is a water decoction prepared by a standardized process according to the theory and clinical application of Chinese medicine, which has the advantages of environmental protection, economy, and clinical applicability. At the same time, it can standardize the clinical use of medication and ensure the accuracy of medication and consistency of dosage (Deng et al., 2019). The pharmacodynamic substances of SGR standard decoction have not been reported. Therefore, we used a comprehensive strategy integrating pharmacodynamic studies and *in vitro* and *in vivo* substance characterization in order to reveal the active components of SGR standard tonics and the mechanism of action for the treatment of HUA.

Chinese medicine has a very ancient history of treating HUA, and its efficacy is remarkable. The treatment approach advocates combining the effects of “dispelling dampness, resolving turbidity, and removing paralysis” to achieve a comprehensive reduction in uric acid levels (Li, et al., 2023). Research also suggests that the “intestine-liver-kidney” axis may be the key organ system involved in uric acid metabolism, representing the primary pathway through which TCM treats hyperuricemia (Lin et al., 2019). From the perspective of pathogenesis and evidence-based TCM treatment, the main therapeutic actions of SGR in treating HUA are to clear heat, detoxify, remove dampness, and alleviate joint discomfort (Hua et al., 2018). So, it is important to find the key target organs and proteins of SGR action *in vivo*. By synthesizing the tissue distribution and molecular docking analysis of SGR active ingredients *in vivo*, the key pathways of SGR standard decoction for HUA treatment can be elucidated more comprehensively. This work intends to integrate chemical fingerprinting, metabolic fingerprinting, and pharmacodynamic fingerprinting analyses to elucidate the active ingredients and mechanism of action of SGR standard decoction for the treatment of hyperuricemia.

**FIGURE 1:**
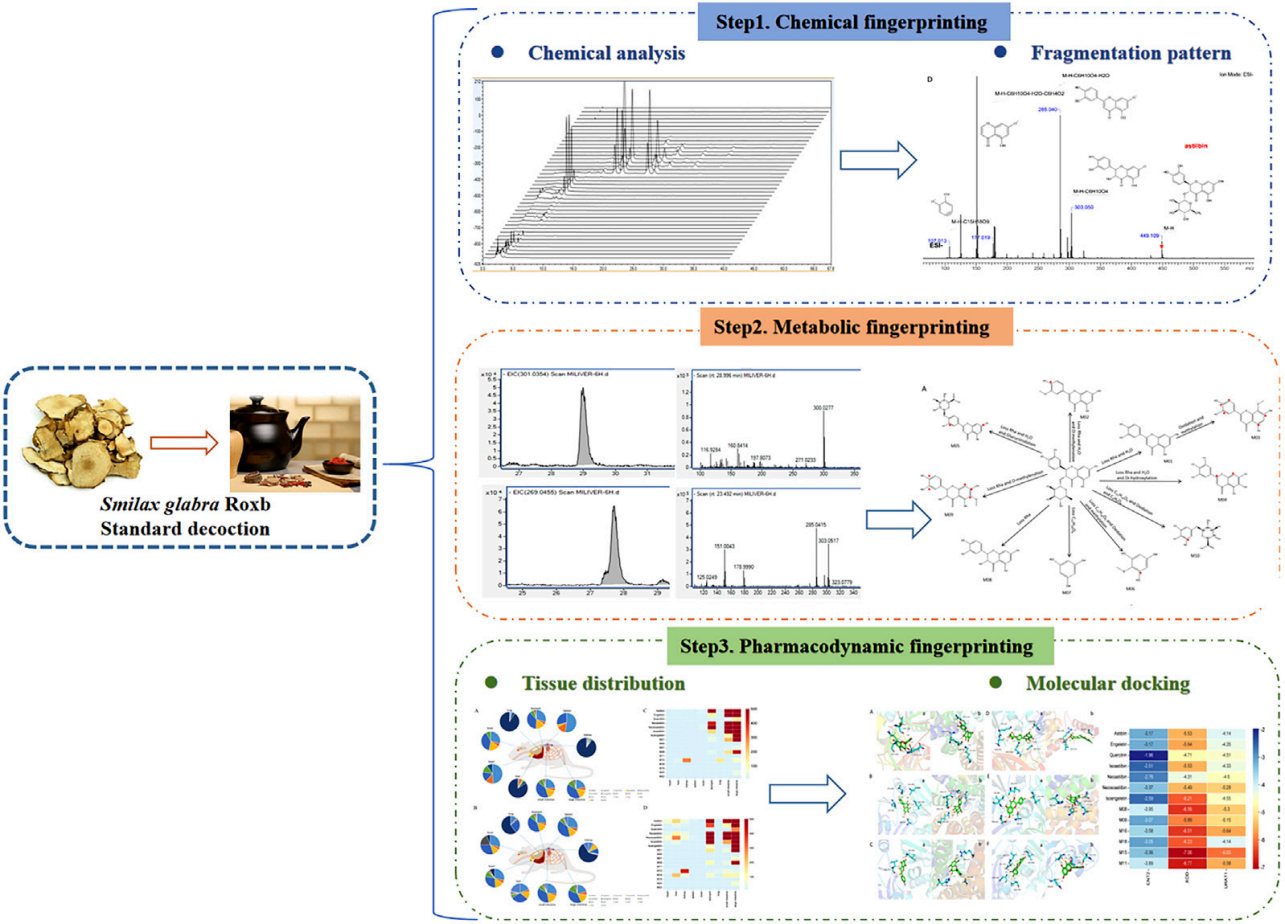
The graphical abstract of the paper.

In the present study, based on the traditional Chinese medicine theory of uric acid reduction, a rapid, highly sensitive, and effective method, UPLC-Q-TOF/MS, was employed to analyze the chemical composition of different polar compound groups in SGR extract. Pharmacodynamic experiments demonstrated the effectiveness of flavonoids in SGR for treating HUA. Additionally, the metabolic fingerprint profile and tissue distribution of the active components in the SGR standard decoction were investigated, and molecular docking simulations were used to explore the binding interactions between key SGR components and uric acid-lowering target proteins. This study provides essential information for identifying the bioactive compounds in SGR and lays the groundwork for future research into their biological properties. Figure 1 presents the holistic view of our research process.

## 2 Materials and methods

### 2.1 Materials and reagents

*Smilax glabra* Roxb was purchased from Shanghai Wanshi Cheng Chinese Medicine Products Co., Ltd. (Shanghai, China). Astilbin (≥93.6%; Batch No. 111798-201805) and Engeletin (93.7%; Batch No. 111906-201102) were obtained from the National Institute for the Control of Pharmaceutical and Biological Products (Beijing, China). Neoastilbin (≥98%; Batch No. B28112), Neoisoastilbin (≥98%; Batch No. B28113), Isoastilbin (≥98%; Batch No. B50661), Quercitrin (≥98%; Batch No. B20526), and Isoengeletin (≥98%; Batch No. B24735) were purchased from Shanghai Yuanye Biotechnology Co., Ltd. (Shanghai, China). Potassium oxonate (purity ≥98.5%, Batch No. SM0510DA13) was sourced from Shanghai Yuanye Biotechnology Co., Ltd. (Shanghai, China). Allopurinol (purity ≥99%, Batch No. 05130302) was obtained from Shanghai Xinyi Wanxiang Pharmaceutical Co., Ltd. (Shanghai, China). Xanthine oxidase was purchased from Nanjing Jiancheng Biotechnology Research Institute (Nanjing, China). HPLC-grade methanol and formic acid were obtained from CNW (Shanghai, China). 95% ethanol was sourced from China National Pharmaceutical Group Chemical Reagent Co., Ltd., and macroporous resins were supplied by Donghong Chemical Co., Ltd. (Anhui, China).

### 2.2 Samples preparation

A 100 g sample of SGR was cut into a fine powder. Then, 900 mL of water was added, and the mixture was soaked for 30 min before being boiled and simmered for 50 min. The solution was immediately filtered, and the residue was extracted with 800 mL of water. This mixture was again boiled and simmered for 40 min, followed by filtration. The two decoctions were then combined and concentrated to 0.2 g/mL under reduced pressure at 60°C.

In the pharmacodynamic study, 25.0 g of D101 resin was wet-packed into a glass column, and 100 mL of the crude SGR extract solution was subjected to chromatography. After sufficient adsorption of the sample, the resin was washed with 2,000 mL of deionized water, followed by successive elution with 10%, 20%, 30%, 40%, 50%, and 95% aqueous ethanol (2,000 mL each). The eluates were analyzed by HPLC, and based on the results, the SGR eluates were grouped into two segments with different polarities: the macro-polar compound group and the medium-polar compound group. Both solutions were concentrated and evaporated to dryness.

### 2.3 Animals and drug administration

Fifteen male Sprague-Dawley (SD) rats, aged 7-8 weeks, were purchased from Shanghai Lingchang Biotechnology Co., Ltd. [Ethical Review No. (TJBH06423101)]. Fifty male Kunming mice, weighing 26–30 g, were obtained from the Shanghai Silaike Animal Experiment Centre [Ethical Review No. (TJBH06424201)]. Both the rats and mice were given 1 week to acclimate, with temperatures maintained at 20°C ± 2°C, a 12-h light/dark cycle, and relative humidity of 50%. The animals were provided with a standard commercial diet and free access to water. The rats were fasted for 10 h prior to drug administration.

In the pharmacodynamic study, 50 Kunming mice were randomly divided into five groups after 1 week of acclimatization: a blank group (NC), a model group (HUA), a Western drug allopurinol group (AP), the SGR large-polar compound group (LPC), and the SGR medium-polar compound group (MPC), with 10 mice in each group. Potassium oxonate, a uricase inhibitor, was used to establish a hyperuricemia model, as it is commonly used to evaluate the uric acid-lowering effects of drugs. The blank group was administered 10 mL/kg of 0.9% saline by gavage, while the other four groups were intraperitoneally injected with 280 mg/kg of 0.5% carboxymethylcellulose sodium (CMC-Na) potassium oxonate suspension. Each day at 14:00, the model, SGR administration, and allopurinol groups were administered potassium oxonate by gavage. One hour later, the SGR high-polarity extract (100 mg/kg), medium-polarity extract (100 mg/kg), and allopurinol aqueous solution (10 mg/kg) were administered by gavage. The normal and model groups received an equivalent volume of saline by gavage once a day for seven consecutive days.

In the metabolic fingerprinting and tissue distribution study, 15 male SD rats were randomly assigned to five groups (n = 3). Blood and tissue samples were collected from groups 1–4 after oral administration of 10 g/kg of SGR complete extract, given twice daily for 3 days. Group 5 served as the blank control, providing baseline biological samples. Systemic blood (400-500 μL) was collected into heparinized tubes at 0.5 h, 1 h, 2 h, and 4 h. Tissue samples (heart, liver, spleen, lungs, kidneys, brain, stomach, small intestine, and large intestine) were collected at 0.5 h and 2 h. Blood samples were centrifuged at 4,000 rpm for 10 min, and tissue samples were homogenized in 0.9% saline.

### 2.4 Biological samples collection and pretreatment

In the metabolic fingerprinting study, approximately 400–500 μL of blood was collected from the retroorbital venous plexus at 0, 1, 2, and 4 h for each group (n = 3). The samples were centrifuged at 4,000 rpm for 10 min to separate the plasma. The supernatant was then dried under nitrogen gas at room temperature. The residue was dissolved in 100 μL of a methanol-water solution (1:9, v/v), and following centrifugation at 13,000 rpm for 10 min, 10 μL of the supernatant was analyzed using UPLC-Q-TOF/MS.

In the pharmacodynamic study, 1 hour after model establishment, 200–300 μL of blood was collected from the retroorbital venous plexus, centrifuged at 4,000 rpm at 4°C for 10 min, and the supernatant was used to determine serum uric acid levels via UV spectrophotometry. Liver and kidney samples (1 g each) were homogenized with 4 mL of normal saline at 60 Hz for 120 s using a tissue grinder. The homogenate was centrifuged at 14,000 rpm at 4°C for 10 min, and the supernatant was used to measure purine oxidase activity in the liver and uric acid levels in kidney tissue following the kit’s instructions.

In the tissue distribution study, 100 mg of tissue was collected from each of nine organs (heart, liver, spleen, lung, kidney, brain, large intestine, small intestine, and stomach). Each sample was homogenized with 1 mL of physiological saline for 120 s using a tissue grinder, then centrifuged at 13,000 rpm at 4°C for 10 min. A 10 μL aliquot of the supernatant was collected for mass spectrometry analysis.

### 2.5 HPLC analysis

HPLC analysis was performed using a high-performance liquid chromatograph (Thermo Fisher Scientific, United States) equipped with a Kromasil C-18 column (4.6 × 250 mm, 5 μm). The mobile phases consisted of 0.1% formic acid in deionized water (A) and methanol (B). The gradient elution was optimized as follows: 0–20 min, 35%–40% B; 20–35 min, 40%–45% B; 35–40 min, 45%–35% B. The flow rate was set to 1 mL/min. The injection volume was 10 μL, and the column temperature was maintained at 30°C. The chromatogram was recorded at 291 nm.

### 2.6 UPLC-Q-TOF/MS analysis

Samples were analyzed using an Agilent 1290 Infinity UPLC system (Milford, MA, United States) equipped with an Agilent EC-C18 poroshell column (2.1 × 150 mm, 1.9 μm). The mobile phase consisted of (A) 0.1% formic acid in water and (B) methanol. The gradient elution was optimized as follows: 0–30 min, 0%–40% B; 30–35 min, 40%–50% B; 35–45 min, 50%–90% B; 45–46 min, 90%–100% B; 46–48 min, 100% B; 48.5 min, 0% B. The flow rate was set at 0.3 mL/min, and the column temperature was maintained at 45°C. The injection volume was 5 μL. An Agilent 6545 Q-TOF-MS/MS system with Agilent Jet Stream electrospray ionization (ESI) was used for detection in negative ion mode. The scan range was 100–1,000 m/z for MS and 50–800 m/z for MS/MS, with a fragment voltage of 175 V. Other parameters included a gas temperature of 320°C, nebulizer gas pressure of 35 psig, sheath gas temperature of 350°C, sheath gas flow of 11 L/min, and drying gas flow of 8 L/min. Calibration during data acquisition was automated. Metabolites were fragmented in MS/MS mode with argon as the collision gas, using collision energies ranging from 10 to 40 eV (low to high).

### 2.7 Data analysis

Agilent MassHunter Qualitative Analysis software (version B.10.00) was used to process full-scan and MS/MS datasets (including all ion and target modes) with a custom-built PCDL library containing known or hypothetical chemical formulas of phytochemical components (mainly flavonoids) and associated metabolites. The chromatogram extraction window was set to 20 ppm, while the parameters for Molecular Feature Extraction (MFE) and Find by Formula (FBF) were a mass error of less than 10 ppm, a peak area greater than 5000 counts, and a maximum of 5 matches. Metabolites were evaluated based on the following criteria: mass error of less than 5 ppm for protonated molecules, consistent isotopic patterns, MS/MS product ions matching parent compounds (using dynamic mass defect filters), and plausible retention times (calculated octanol/water partition coefficient using Clog P).

### 2.8 Molecular docking analysis

The interactions between astilbin, engeletin, quercitrin, isoastilbin, neoastilbin, neoisoastilbin, isoengeletin, and metabolites M08, M09, M16, M18, M15, and M11 with CNT2, XOD, and URAT1 were simulated using AutoDock Vina software to investigate a potential binding model. The molecular structures of these compounds were retrieved from the PubChem database (https://pubchem.ncbi), while the crystal structures of XOD (PDB ID: 1FIQ) and NLRP3 (PDB ID: 6NPY) were downloaded from the RCSB PDB database (http://www.rcsb.org/). Homology modeling was performed to construct the CNT2 structure using the SWISS-MODEL web server (http://swissmodel.expasy.org). After removing water molecules and original ligands using PyMOL 2.3.0, CNT2, XOD, and URAT1 were optimized using Chem3D (2020 version) software. The pretreated proteins were hydrogenated and torsion bonds were determined using AutoDock Tools 1.5.6. Docking was performed in the active site pockets of the target proteins, and the results were visualized using PyMOL 2.3.0.

## 3 Results

### 3.1 Separation of ingredients in SGR by macroporous resins

**FIGURE 2:**
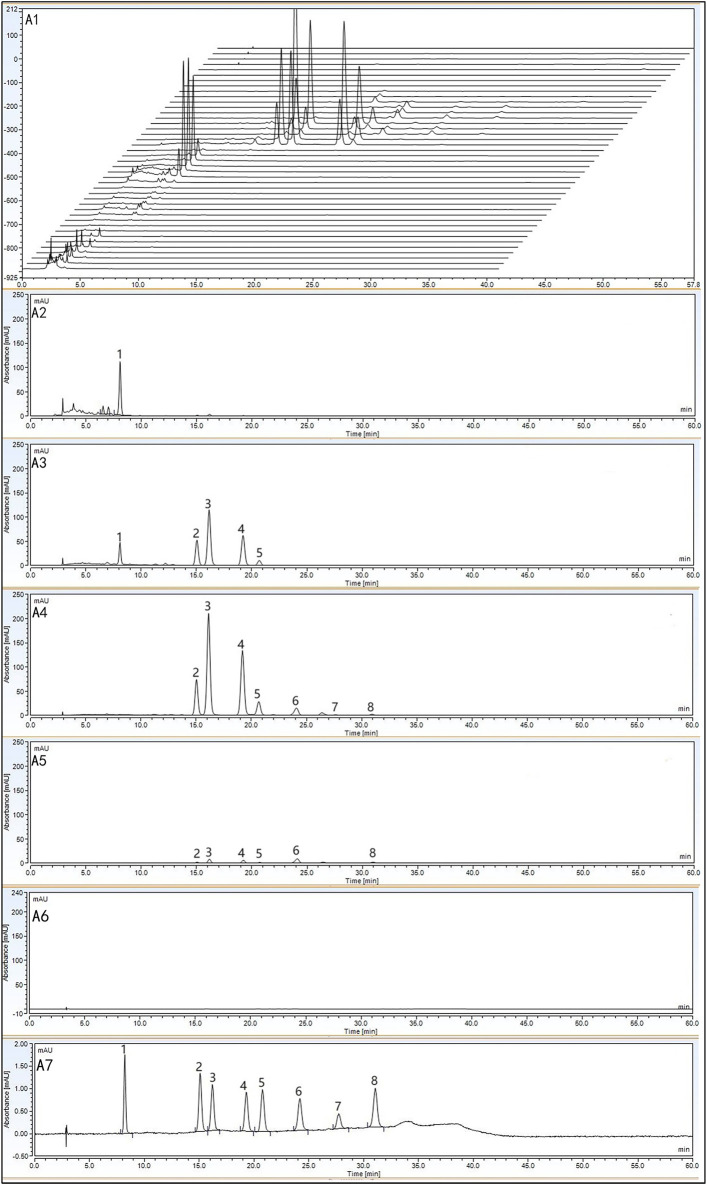
HPLC chromatograms of 10%, 20%, 30%, 40%, 50%, and 95% ethanol eluates and reference standards: Chromatograms of all ethanol eluates **(A1)**; 10% ethanol eluate **(A2)**; 20% ethanol eluate **(A3)**; 30% ethanol eluate **(A4)**; 40% ethanol eluate **(A5)**; 50% ethanol eluate **(A6)**; reference standards **(A7)**.

Previous experimental studies identified flavonoids in SGR as potential quality markers for its uric acid-lowering pharmacodynamic effects (Shi et al., 2023), although the pathways through which these flavonoids exert their effects *in vivo* remain unclear. To address this, we used macroporous resin to fractionate the components in SGR and conducted a pharmacodynamic study focused on uric acid-lowering activity. Based on a literature review and the adsorption characteristics of resins, D101 macroporous resin was selected for the experiments (Su et al., 2022). The components in each group of eluates were initially identified using HPLC chromatography and standard comparisons. The constituents of the 10%, 20%, 30%, 40%, 50%, and 95% aqueous ethanol (v/v) eluates of SGR were analyzed, and their chromatograms are shown in Figure 2A1. The 10% ethanol eluent primarily contained 5-o-caffeoylshikimic acid (Figure 2A2). The 20% ethanol eluent mainly comprised 5-o-caffeoylshikimic acid, neoastiblin, astilbin, neoisoastiblin, and isoastiblin (Figure 2A3). The 30% ethanol eluent primarily included neoastiblin, astilbin, neoisoastiblin, isoastiblin, engeletin, quercitrin, and isoengeletin (Figure 2A4). The 40% ethanol eluent contained primarily neoastiblin, astilbin, neoisoastiblin, isoastiblin, engeletin, and isoengeletin (Figure 2A5). The 50% ethanol eluent contained virtually no components (Figure 2A6). Figure 2A7 displays the chromatograms of the reference standards. Since the 20%, 30%, and 40% ethanol eluates predominantly contained flavonoids, they were combined and referred to as the medium-polar compound group. The 10% ethanol eluent was designated as the large-polar compound group. Each polar eluent was concentrated and evaporated at 60°C under reduced pressure to obtain the large-polar compound group of SGR (10% ethanol eluent) and the medium-polar compound group of SGR (20%, 30%, and 40% ethanol eluents).

### 3.2 Characterisation of different polar component groups in SGR by UPLC-Q-TOF/MS

**FIGURE 3:**
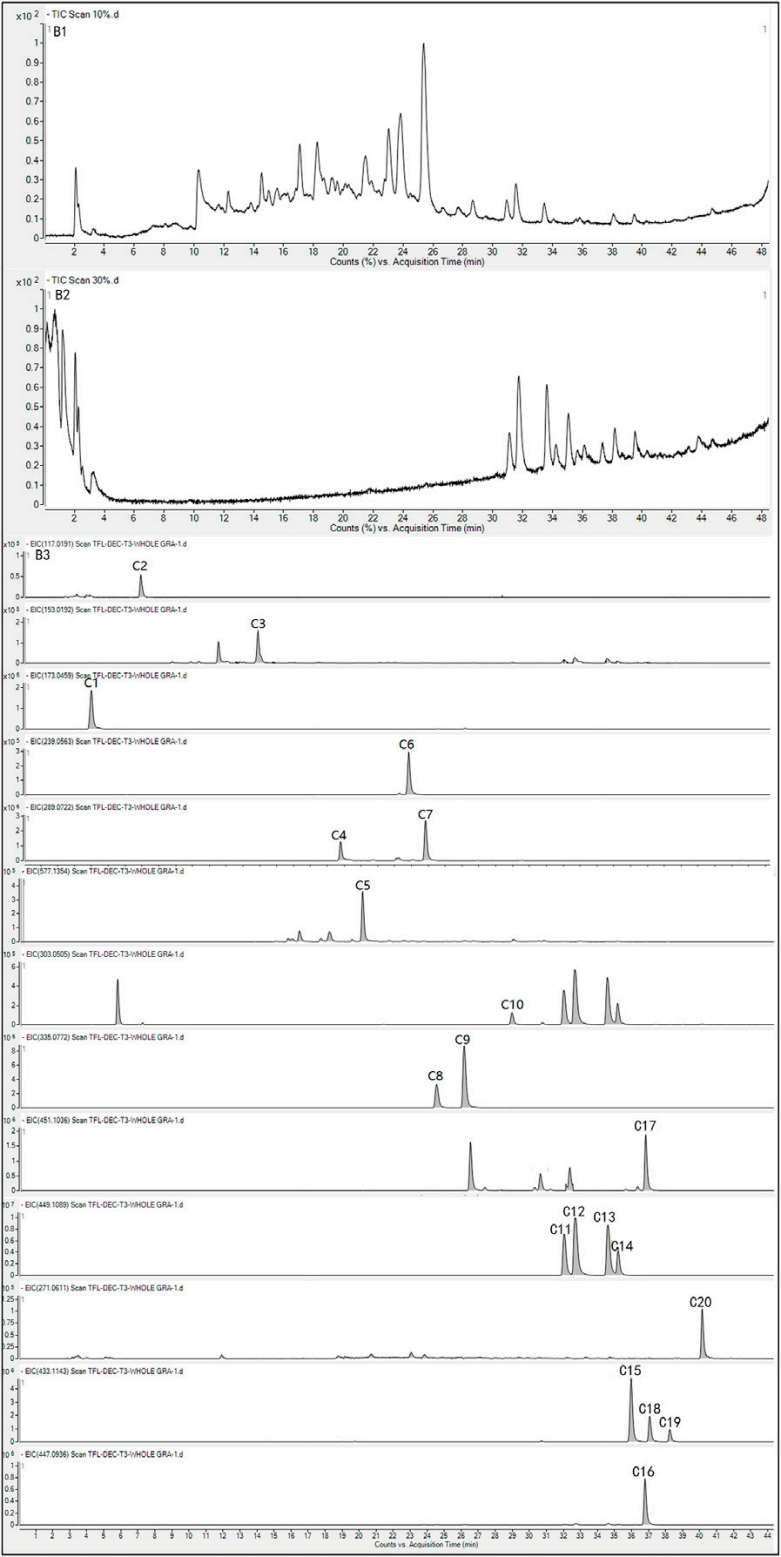
TIC **(B1)** and EIC **(B3)** of components in the large-polar group of SGR in negative mode; TIC **(B2)** and EIC **(B3)** of components in the medium-polar group of SGR in negative mode.

To further characterize the components within the large and medium polar compound groups, UPLC-Q-TOF/MS was employed. The results are shown in Figure 3B. To build an ingredient library for SGR, ChemSpider was used to summarize and analyze 20 compounds, including their names, molecular formulas, exact molecular weights, and chemical structures. Chromatographic and spectrometric data from unknown compounds were tentatively identified based on previous literature using UPLC-Q-TOF/MS. For known compounds, their structures were tentatively determined by comparison with reference standards.

Total ion chromatograms (TIC) and extracted ion chromatograms (EIC) for the large-polar compound group (Figures 3B1, 3B3) and the medium-polar compound group (Figures 3B2, 3B3) in negative mode were obtained. Extracted ion chromatograms of 20 compounds derived from SGR and reference standard solutions were analyzed using UPLC-Q-TOF/MS in ESI mode (Table 1). In addition to the [M-H]- ions, the phenolic hydroxyl groups in the core structure of the flavonoids produced [M-H-H_2_O]- ions. Figure 2B1 shows the components of the large-polar compound group, which included six organic acids and three flavonoids. Figure 2B2 displays the components in the medium-polar compound group, consisting of 11 flavonoids. Thus, macroporous resin effectively separated the active components of SGR, and we concentrated the two polar fractions separately for pharmacodynamic testing.

**TABLE 1:** Characterization of constituents of SGR by UPLC-Q-TOF/MS.

ID	Name	Formula	RT (min)	Adduct	MS/MS(m/z)	Error (ppm)
				[M-H]^-^		
C1	Shikimic acid	C_7_H_10_O_5_	4.01	173.0459	155, 133, 111	2.31
C2	succinic acid	C_4_H_6_O_4_	6.94	117.0191	112, 103	1.7
C3	protocatechuic acid	C_7_H_6_O_4_	13.9 -	153.0192	141, 119, 109	6.53
C4	Catechin	C_15_H_14_O_6_	18.79	289.0722	179, 151, 137	1.38
C5	procyanidin B	C_30_H_26_O_12_	20.19	577.1354	451, 289, 137	5.19
C6	Syringic acid acetate	C_11_H_12_O_6_	22.84	239.0563	218, 195, 179	8.36
C7	epicatechin	C_15_H_13_O_6_	23.83	289.0722	179, 151, 137	2.98
C8	4-o-caffeoylshikimic acid	C_16_H_16_O_8_	24.55	335.0772	291, 179, 135	1.19
C9	5-o-caffeoylshikimic acid	C_16_H_16_O_8_	26.16	335.0772	291, 179, 135	2.98
C10	cinchonain Ia	C_24_H_20_O_9_	30.66	451.1036	433,341, 303	2.22
C11	neoastiblin	C_21_H_22_O_11_	32.04	449.1089	303, 285, 177	−1.11
C12	astilbin	C_21_H_22_O_11_	32.69	449.1089	303, 285, 177	2.22
C13	neoisoastiblin	C_21_H_22_O_11_	34.61	449.1089	303, 285, 177	−2.22
C14	isoastiblin	C_21_H_22_O_11_	35.22	449.1089	303, 285, 177	−1.11
C15	engeletin	C_21_H_22_O_10_	35.99	433.1143	339, 287, 269	4.61
C16	quercitrin	C_21_H_20_O_11_	36.79	447.0936	343, 301	6.71
C17	cinchonain Ib	C_24_H_20_O_9_	36.88	451.1036	433,341,303	4.43
C18	neoisoengeletin	C_21_H_22_O_10_	37.07	433.1143	339, 287, 269	4.61
C19	isoengeletin	C_21_H_22_O_10_	38.25	433.1143	339, 287, 269	6.92
C20	naringenin	C_15_H_12_O_5_	40.15	271.0611	177, 151, 125	−3.68

**FIGURE 4:**
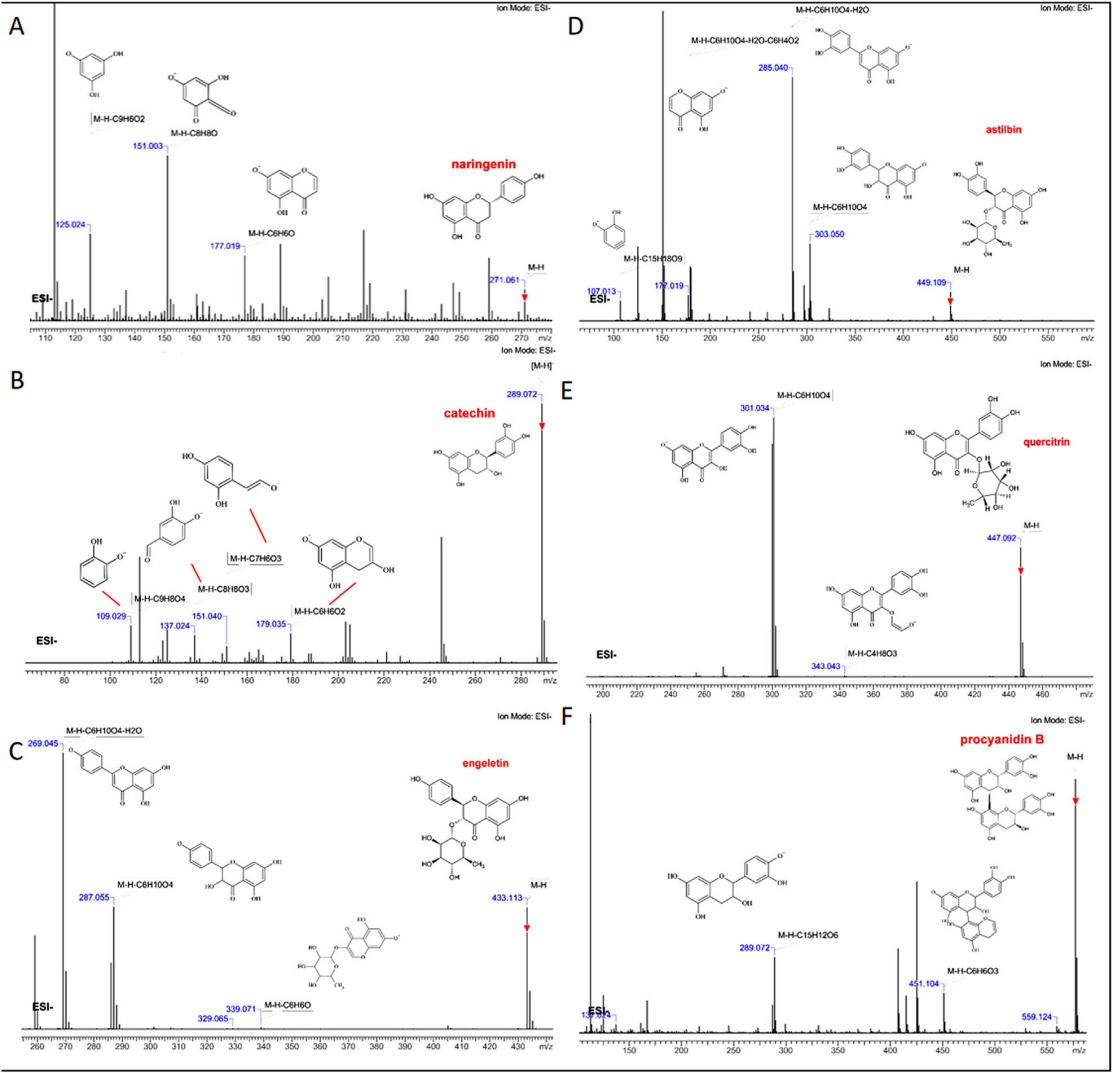
Fragment interpretation of six absorbed flavonoids **(A)** naringenin, **(B)** catechin, **(C)** engeletin, **(D)** quercitrin, **(E)** astilbin, and **(F)** procyanidin B.

The retention time of the detected flavanonol C4 was 19.02 min. Catechin displayed an [M-H]- ion at m/z 289.0718, with a mass error of 1.38 ppm, automatically matching catechin compounds in a self-established database (Figure 4A). In the MS/MS spectrum, fragment ions with high m/z abundances at 179, 151, 137, and 109 were observed. Based on fragment interpretation, the ion at m/z 179 resulted from the loss of [M-H-C_6_H_6_O_2_]-. The ion at m/z 151 was generated by the loss of C_7_H_6_O_3_ from the m/z 289 ion. The ion at m/z 137 was formed due to the loss of [M-H-C_8_H_8_O_3_]-, and the ion at m/z 109 was produced by the loss of C_9_H_8_O_4_ from m/z 289. The retention time of the detected flavanonol C5 was 20.19 min. A [M-H]- ion at m/z 577.1351 was detected for procyanidin B, with the formula C_30_H_26_O_12_ and a mass error of 5.19 ppm, corresponding to procyanidin B compounds in the self-established database (Figure 4B). In the MS/MS spectrum, fragment ions with high m/z abundances at 451, 289, and 137 were identified. The ion at m/z 451 was formed by the loss of C_6_H_6_O_3_ from m/z 577. The fragment ion at m/z 289 resulted from the loss of C_15_H_12_O_6_ from m/z 577, and the ion at m/z 137 was generated by the loss of C_8_H_8_O_3_ from m/z 289.

The retention times of the detected flavonoids C11-C14 were 32.04, 32.69, 34.61, and 35.22 min, respectively. The deduced molecular formulas for these flavonoids were all C_21_H_22_O_11_, with mass errors of −1.11, 2.22, −2.22, and −1.11 ppm, which automatically matched neoastiblin, astilbin, neoisoastiblin, and isoastiblin compounds from the self-established database (Figure 4C). Astilbin, at m/z 449.1089, showed [M-H]- along with its three isomers, all sharing the molecular formula C_21_H_22_O_11_. In the MS/MS spectra, all four compounds generated fragment ions with m/z values at 303, 285, 177, and 107. Based on fragment interpretation, the ion at m/z 303 was formed by the loss of the rhamnoside moiety [M-H-Rha]-. The fragment at m/z 285 indicated the loss of H_2_O. The ion at m/z 177 resulted from the loss of C_6_H_4_O_2_ from m/z 285. The fragment ion at m/z 107 was generated by the loss of [M-H-C_15_H_18_O_9_]-. The retention times of the detected flavonoids C15, C18, and C19 were 35.99, 37.07, and 38.25 min, respectively. The molecular formula for engeletin and its isomer was C_21_H_22_O_10_, with [M-H]- detected at m/z 433.1140. Mass errors of 4.61, 4.61, and 6.92 ppm, respectively, automatically matched engeletin, neoisoengeletin, and isoengeletin compounds from the self-established database (Figure 4D). Both compounds generated fragment ions with m/z values at 339, 287, and 269 in the MS/MS spectra. The ion at m/z 339 resulted from the loss of C_6_H_6_O from m/z 433. The [M-H-Rha]- fragment at m/z 287 was produced by the loss of the rhamnoside moiety. The ion at m/z 269 indicated the loss of H_2_O from m/z 287. The retention time of the detected flavanonol C16 was 36.79 min. Quercitrin exhibited an [M-H]- ion at m/z 447.0933, with a mass error of 6.71 ppm, corresponding to quercitrin in the self-established database (Figure 4E). In the MS/MS spectrum, fragment ions with high m/z abundances at 343 and 301 were observed. According to fragment interpretation, the ion at m/z 343 was formed by the loss of [M-H-C_4_H_8_O_3_]-. The fragment ion at m/z 301 resulted from the loss of the rhamnoside moiety [M-H-Rha]-. The retention time of the detected flavonoid C20 was 40.15 min. Naringenin, with the formula C_21_H_22_O_10_, showed an [M-H]- ion at m/z 433.1140, with a mass error of −3.68 ppm, corresponding to naringenin in the self-established database (Figure 4F). In the MS/MS spectra, ions with high abundances were observed at m/z 177, 151, and 125. The ion at m/z 177 was formed by the loss of C_6_H_6_O from m/z 271. The fragment ion at m/z 151 resulted from the loss of [M-H-C_8_H_8_O]-. The ion at m/z 125 was generated by the loss of C_9_H_6_O_2_ from m/z 271. Thus, by characterizing the chemical fingerprints of SGR, it is possible to identify the components with different polarities, and flavonoids with the same parent nucleus structure exhibit similar cleavage patterns.

### 3.3 Pharmacodynamic study

#### 3.3.1 Replication of hyperuricemia mice model

Allopurinol is currently the most commonly used drug for the treatment of hyperuricemia, as it inhibits the synthesis of endogenous purines and reduces uric acid production (Zhang et al., 2019). Therefore, allopurinol was selected as the positive control drug in this study. Since uricase is present in both mice and rats, it readily breaks down uric acid into the water-soluble allantoin, which is excreted in the urine. This makes it more challenging to sustain uric acid accumulation in these animals, thus complicating the replication of a persistent hyperuricemia model. To address this, uricase inhibitors were used to replicate the model, and an acute hyperuricemia model was established in mice by monitoring changes in blood uric acid levels following intraperitoneal injection of potassium oxonate (Hou et al., 2023), this approach allowed us to investigate the pharmacological basis for the blood uric acid-lowering effects of SGR.

Ten male Kunming mice were intraperitoneally injected with potassium oxonate at 280 mg/kg, and blood samples (300–400 µL) were collected from the retro-orbital venous plexus at 0, 0.5, 1, and 1.5 h post-injection. Absorbance was measured at 690 nm using an ultraviolet spectrophotometer, and serum uric acid levels were calculated using Equation 1.

$$C_{S}\;\left(µ\text{mol}/L\right)=\frac{{\text{OD}}_{sample}}{{\text{OD}}_{standard}}\times C_{standard}$$ (1)

$C_{S}$ : Serum uric acid values; ${\text{OD}}_{sample}$ : the optical density of samples; ${\text{OD}}_{standard}$ : the optical density of standards; $C_{standard}$ : Concentration of standard solution.

**FIGURE 5:**
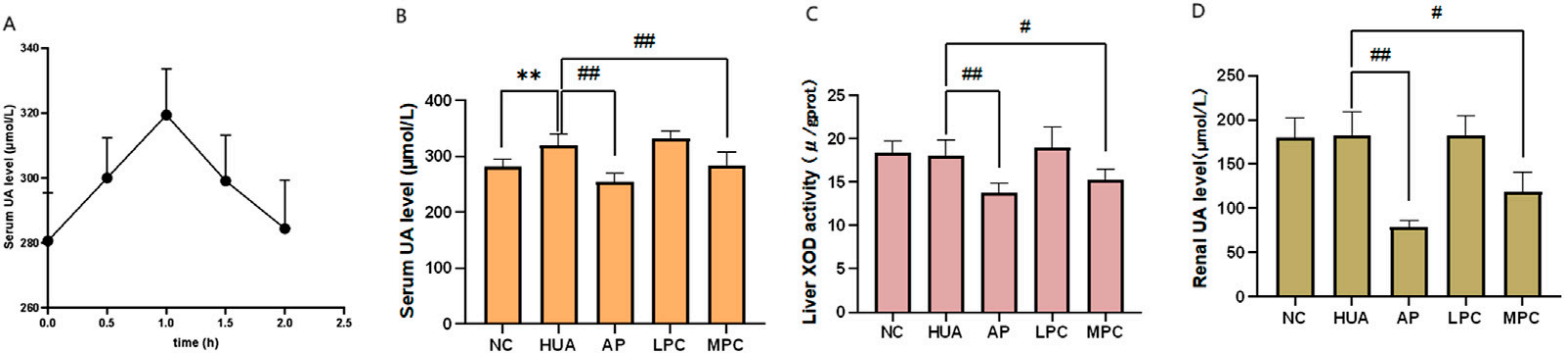
Effects of compound groups on serum urate levels, xanthine oxidase (XOD) activity, and renal uric acid values in mice treated with potassium oxonate. **(A)** Serum uric acid values at various time points after intraperitoneal injection of potassium oxonate in mice; **(B)** Serum uric acid values in each compound group after 1 h of modeling; **(C)** Liver xanthine oxidase (XOD) activity in mice from each compound group; **(D)** Renal uric acid values in each compound group after 1 h of modeling.

The results of the serum uric acid experiments at various time points after the potassium oxonate injection (Supplementary Table S1, Figure 5A) indicated that serum uric acid levels in mice initially increased and then decreased, peaking at 1 h post-injection. This increase was significantly different from the pre-injection levels (*p* < 0.01). After 1 h, serum uric acid levels gradually returned to baseline. These findings suggest that intraperitoneal injection of potassium oxonate can induce hyperuricemia in mice over a short period. Therefore, we chose to evaluate the efficacy of each polar compound group of *SGR* 1 h after modeling.

#### 3.3.2 Determination of serum uric acid in mice

In clinical practice, changes in serum uric acid levels are a key indicator for monitoring the condition of HUA patients, guiding medication use, and assessing the efficacy and prognosis of complications (Li et al., 2023). Therefore, measuring serum uric acid content is the most commonly used index in clinical settings, playing a significant role in evaluating HUA. Serum uric acid values were calculated according to Equation 1, and the results are presented in Supplementary Table S2 and Figure 5B.

Serum uric acid levels were significantly elevated in the model group compared to the blank group (*p* < 0.01), confirming successful model replication. In comparison to the model group, serum uric acid levels decreased in both the allopurinol group and the SGR medium-polar compound group, with the difference between these groups and the model group being significant (*p* < 0.05). However, there was no significant difference between the allopurinol group and the SGR medium-polar compound group (*p* > 0.05), indicating that the SGR medium-polar compound group had a similar inhibitory effect on hyperuricemia induced by potassium oxonate as allopurinol. In contrast, no statistically significant difference (*p* > 0.05) was observed between the SGR large-polar compound group and the model group, suggesting that the large-polar compound group of SGR did not have a notable effect on reducing blood uric acid levels in mice.

#### 3.3.3 Determination of xanthine oxidase (XOD) activity in mice liver

Xanthine oxidase (XOD) activity in mouse liver was measured at 508 nm using a spectrophotometer, following the addition of samples and procedures according to the kit instructions. Absorbance values were recorded at 508 nm, with the results presented in Supplementary Table S3 and Figure 5C. The formula for calculating XOD activity is provided in Equation 2.

$$\text{XOD activity}\left(U/g\right)=\frac{{\text{OD}}_{sample}-{\text{OD}}_{blank}}{\text{Molar}\;\text{extinction}\;\text{coefficient}}\times \frac{V_{Total}}{V_{sample}}\times \frac{1}{\text{Optical}\;\text{path}\times \text{Reaction}\;\text{time}}\;C_{\;Protein}$$ (2)

${\text{OD}}_{sample}$ : the optical density of samples; ${\text{OD}}_{standard}$ : the optical density of blank; $V_{Total}$ : total volume of reaction solution; ${\;V}_{sample}$ : volume of sample solution; $C_{Protein}$ : homogenized protein concentration.

The molar extinction coefficient of the liver sample was 12.6 × 10^3^, the total reaction volume was 2.29 mL, the sample volume was 20 μL, the optical path length was 1 cm, the reaction time was 20 min, and the protein concentration in the homogenate was 19.32 g/L (1:4 homogenate). As shown in Figure 5C, the XOD levels in the blank group and the model group were similar, with no statistically significant difference between the two. This may be due to the short modeling time in the model group, as the modeling mechanism involved inhibiting uricase catabolism rather than inhibiting xanthine oxidase activity, thus having minimal impact on liver XOD levels. In contrast, XOD levels in both the allopurinol group and the medium-polar compound group were reduced. There was a highly significant difference between the allopurinol group and the model group (*p* < 0.01) and a significant difference between the medium-polar compound group and the model group (*p* < 0.05), but no significant difference between the medium-polar compound group and the allopurinol group (*p* > 0.05). Allopurinol, a standard treatment in Western medicine, inhibits uric acid production by suppressing XOD activity, thereby preventing the conversion of xanthine and hypoxanthine into uric acid. This suggests that the medium-polar compound group of *SGR* was able to lower blood uric acid levels in the HUA model mice, possibly through a mechanism similar to allopurinol, by inhibiting XOD activity and reducing uric acid production. In contrast, there was no statistically significant difference between the SGR large-polar compound group and the model group, indicating that the large-polar compound group had no inhibitory effect on hepatic XOD activity. This could explain why it did not reduce blood uric acid levels in the HUA mice.

#### 3.3.4 Determination of uric acid in mice kidney

Renal uric acid values were calculated using Equation 1. Supplementary Table S4 and Figure 5D show the renal uric acid levels in mice from each group. The renal uric acid values of the model group, the SGR large-polar compounds group, and the blank group were similar, with no statistically significant differences between these groups and the blank group (*p* > 0.5). This suggests that although the HUA model, induced by a one-time intraperitoneal injection of potassium oxonate, increased serum uric acid levels, it did not affect uric acid levels in the kidneys. This further indicates that the large-polar compounds group of SGR did not inhibit uric acid levels in the kidneys. In contrast, both the allopurinol group and the SGR medium-polar compound group showed reduced uric acid levels in the kidneys of mice. There was a highly significant difference between the allopurinol group and the model group (*p* < 0.01) and a significant difference between the SGR medium-polar compound group and the model group (*p* < 0.05). These findings suggest that, similar to allopurinol, the SGR medium-polar compound group effectively reduced uric acid levels in the kidneys.

In summary, the pharmacodynamic results suggest that the medium-polar compounds in SGR may lower uric acid levels through two mechanisms: promoting the excretion of uric acid via the kidneys and inhibiting xanthine oxidase (XOD) activity in the liver, thereby reducing uric acid production.

### 3.4 Identification of absorbed flavonoids from SGR

**FIGURE 6:**
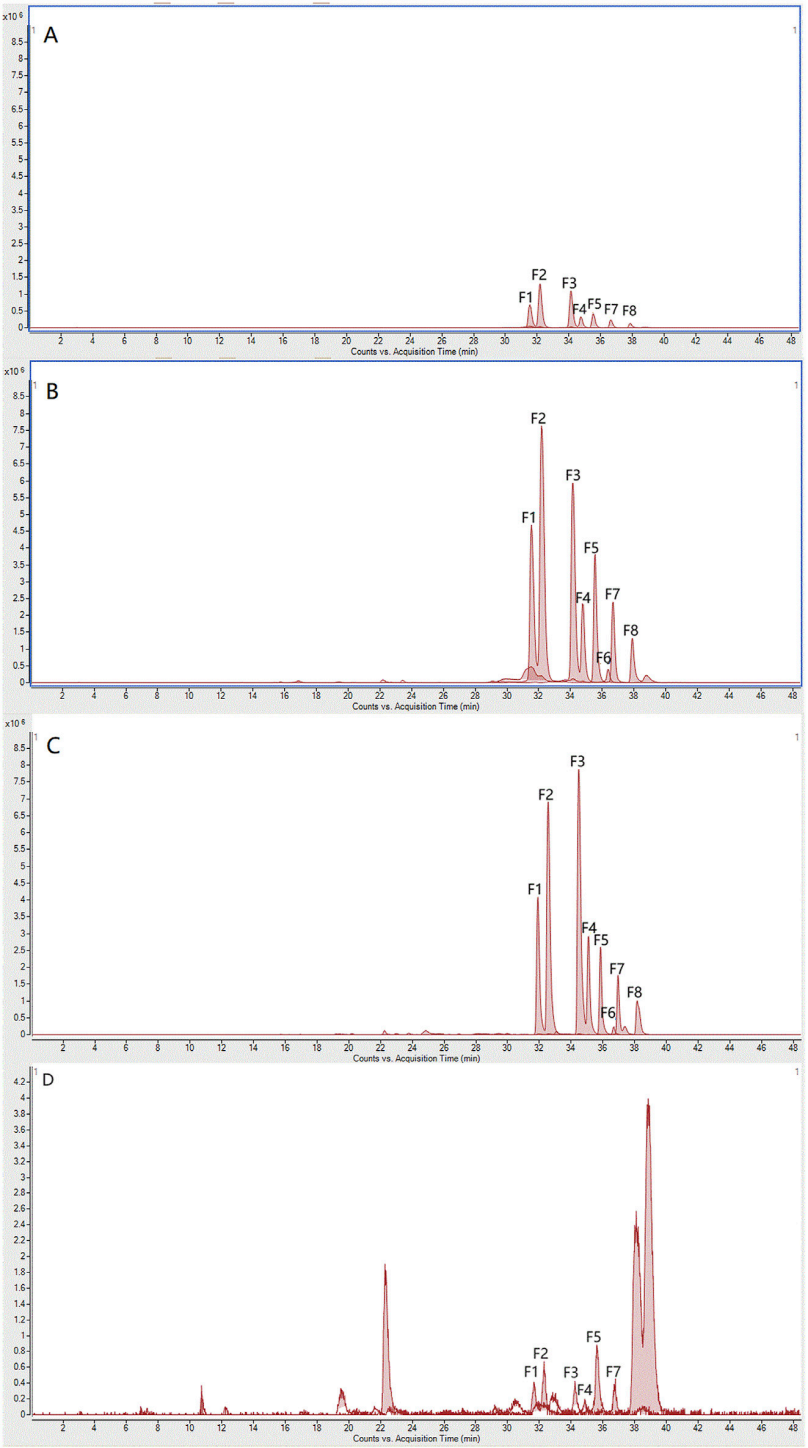
Ion chromatograms after oral administration of absorbed flavonoids from SGR at **(A)** 0.5 h, **(B)** 1 h, **(C)** 2 h, **(D)** 4 h.

A total of eight flavonoids were detected *in vivo* following oral administration of SGR extract, with these compounds also being present at relatively high levels *in vitro*. We monitored the changes in the eight components *in vivo* over time at 30 min, 1 h, 2 h, and 4 h. Due to the complex composition of plasma samples, background subtraction (blank plasma) and baseline calibration were performed first. Figures 6A–D show the chemical structures and ion chromatograms of the identified absorbed dihydroflavonols F1-F8 at 30 min, 1 h, 2 h, and 4 h. By utilizing literature and public databases, we identified the compounds in rat plasma by comparing their molecular formulas, fragment ions, and chemical structures. Based on the measurements of the plasma samples over the four time points, it was clear that the flavonoids were well separated and exhibited distinct differences in content. Table 2 presents the complete identification results. The flavonoids F1-F8 share the same core structure, that is, the 2-phenylchromogenic ketone structure, with varying substitution positions. The predominant groups include substituted methoxy and hydroxyl groups. Under the same collision-induced dissociation conditions, the flavonoids exhibited similar fragment ions and corresponding relative intensities. These findings indicate that flavonoids are the primary active components absorbed from SGR into the bloodstream.

**TABLE 2:** Identification of absorbed flavonoids in rat plasma by UPLC-Q-TOF/MS.

ID	Name	Formula	RT (min)	Adduct	MS/MS(m/z)	dM(ppm)
				[M-H]-		
F1	neoastiblin	C_21_H_22_O_11_	31.93	449.1089	303, 285, 177	−1.11
F2	astilbin	C_21_H_22_O_11_	32.56	449.1089	303, 285, 177	2.22
F3	neoisoastiblin	C_21_H_22_O_11_	34.51	449.1089	303, 285, 177	−2.22
F4	isoastiblin	C_21_H_22_O_11_	35.14	449.1089	303, 285, 177	−1.11
F5	engeletin	C_21_H_22_O_10_	35.89	433.1143	339, 287, 269	4.61
F6	quercitrin	C_21_H_20_O_11_	36.70	447.0936	343, 301	6.71
F7	neoisoengeletin	C_21_H_22_O_10_	36.99	433.1143	339, 287, 269	4.61
F8	isoengeletin	C_21_H_22_O_10_	38.19	433.1143	339, 287, 269	6.92

### 3.5 Metabolic profile of absorbed flavonoids in plasma

**FIGURE 7:**
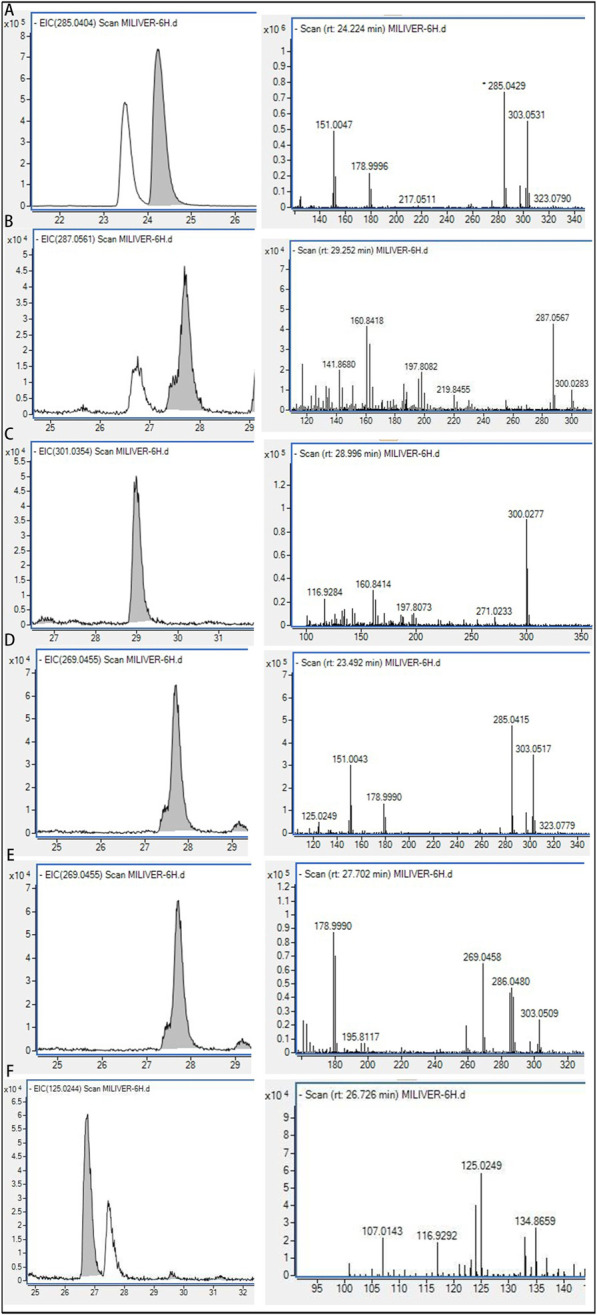
EIC spectrogram and fragmentation pattern of five metabolites in rats. **(A)** M4; **(B)** M6; **(C)** M8; **(D)** M11; **(E)** M15; (F) M20.

**FIGURE 8:**
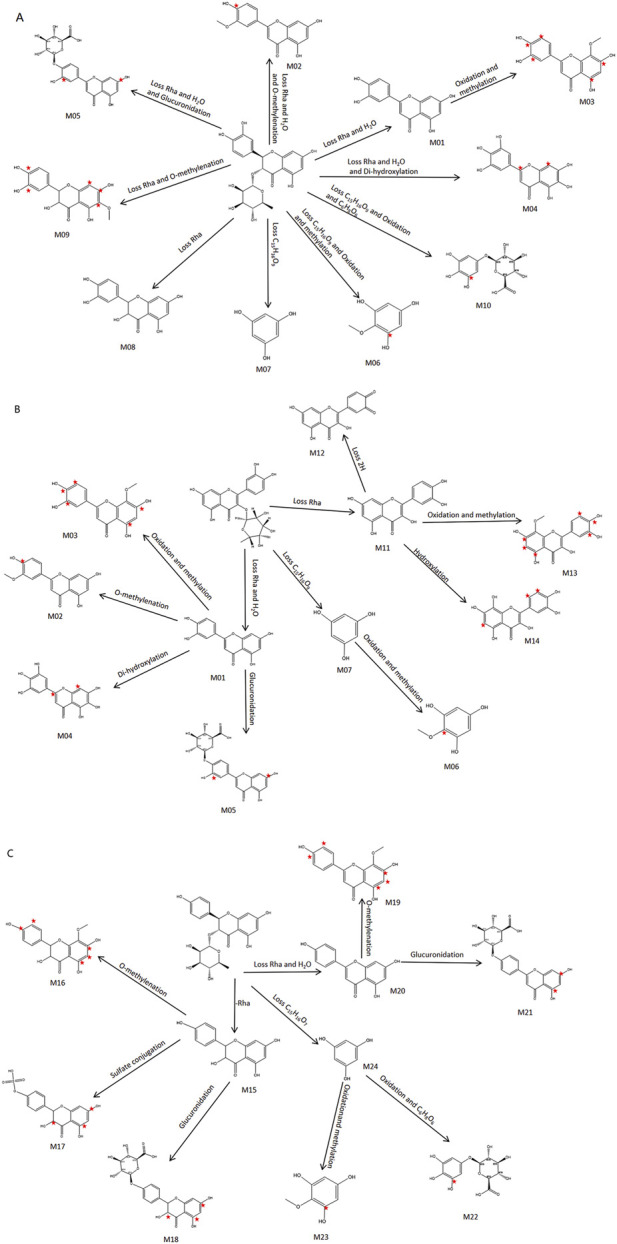
Proposed major metabolic pathway of **(A)** astilbin, **(B)** quercitrin, **(C)** engeletin.

Cleavage patterns are generally similar between parent compounds and their metabolites (Wu et al., 2022b). Based on the fragmentation patterns of the standards, we analyzed the cleavage characteristics and metabolites of structurally similar compounds. By combining retention times, clog P values, and differences at various time points, compounds with identical structures were excluded (Li et al., 2020). This method allows for more comprehensive detection of rare chemical metabolites. As is well known, the major phase I reactions include hydroxylation, dealkylation, hydrolysis, and oxidation, while phase II reactions include sulfate and glutamate conjugation, among others (Cui et al., 2023). From the plasma fragmentation patterns of astilbin, quercitrin, and engeletin, 24 metabolites (including indistinguishable isomers) were identified, labeled as M1-M24: M1-M10 and M15-M24 were metabolites derived from astilbin and engeletin, while M1-M7 and M11-M14 were metabolites derived from quercitrin. Extracted ion chromatograms for some metabolites are shown in Figure 7. Detailed MS/MS fragmentation, identification results, and potential metabolic pathways are presented in Figure 8 and Table 3.

**TABLE 3:** Summary of SGR metabolites in rat plasma.

Met	Biotransformation	Formula	[M−H]− (m/z)	RT min	MS/MS fragments	Parent
M01	Loss Rha and H2O	C15H1006	285.0404	31.683,32.332,34.313,34.912	259,179,151	astilbin, quercitrin
M02a	Loss Rha and H2O and O-methylenation	C15H807	299.0197	31.516	285,193,155	astilbin, quercitrin
M03abcdef	Loss Rha and H2O and Oxidation and methylation	C16H1207	315.051	15.701	289,271,242	astilbin, quercitrin
M04abc	Loss Rha and H2O and Di-hydroxylation	C15H1008	317.0302	21.561	289,212,193	astilbin, quercitrin
M05abc	Loss Rha and H2O and Glucuronidation	C21H18012	461.0725	19.513	449,341,303	astilbin, quercitrin
M06	Loss C15H16O9 and Oxidation and methylation	C16H12O7	155.035	32.565	151,137,107	astilbin, quercitrin
M07	Loss C15H16O9	C6H6O3	125.0244	31.683,32.332,34.313,34.912	116,112,107	astilbin, quercitrin
M08	Loss Rha	C15H1207	303.0510	31.683,32.332,34.313,34.912	285,151,125	astilbin
M09abcdef	Loss Rha and O-methylenation	C16H1408	333.0409	5.788	291,179,161	astilbin
M10	Loss C15H16O9 and Oxidation and C6H8O6	C12H14O10	317.0656	33.535	229,185,141	astilbin
M11	Loss Rha	C15H1007	301.0353	36.511	287,248,175	quercitrin
M12	Loss Rha and loss 2H	C15H8O7	299.0197	36.511	287,271,192	quercitrin
M13abcdefg	Loss Rha and and Oxidation and methylation	C16H12O8	331.0459	9.807	301,269,221	quercitrin
M14abcdef	Loss Rha and Hydroxylation	C12H14O10	317.0514	36.271	299,287,269	quercitrin
M15abc	Loss Rha	C15H12O6	287.0561	35.695,36.810,38.009	269,151,107	engeletin
M16abcdef	Loss Rha and O-methylenation	C16H14O7	317.0666	22.976	287,179,135	engeletin
M17abcd	Loss Rha and Sulfate conjugation	C15H12O9S	367.0129	23.292	339,303,289	engeletin
M18abcd	Loss Rha and Glucuronidation	C21H20O12	463.0882	35.828,37.093,38.375	433,317,299	engeletin
M19abcdef	Loss Rha and H2O and O-methylenation	C16H12O6	299.0561	35.861,37.110,38.392	269,179,116	engeletin
M20	Loss Rha and H2O	C15H10O5	269.0455	35.695,36.810,38.009	175,151,107	engeletin
M21abc	Loss Rha and H2O and Glucuronidation	C21H18O11	445.0776	16.459	429,380,225	engeletin
M22	Loss C15H16O7 and Oxidation and C6H8O6	C12H14O10	317.0656	35.845	299,269,178	engeletin
M23	Loss C15H16O7 and Oxidation and methylation	C7H8O4	155.035	31.350	151,137,125	engeletin
M24	Loss C15H16O7	C6H6O3	125.0244	35.755	116,112,107	engeletin

For astilbin, a total of 10 metabolites (M1-M10) were identified. The observed biotransformations included: loss of Rha and loss of H_2_O (M1); loss of Rha, H_2_O, and O-methylenation (M2); loss of Rha, H_2_O, oxidation, and methylation (M3); loss of Rha, H_2_O, and di-hydroxylation (M4); loss of Rha, H_2_O, and glucuronidation (M5); loss of C_15_H_12_O_9_, oxidation, and methylation (M6); loss of C_15_H_12_O_9_ (M7); loss of Rha (M8); loss of Rha and O-methylenation (M9); and loss of C_15_H_16_O_9_, oxidation, and C_6_H_8_O_6_ (M10). Astilbin was detected in plasma samples in high abundance 2 h after oral administration. The retention times of the metabolites ranged from 20.14 to 40.12 min, with the parent compound eluting after 32.35 min. Figure 8A illustrates the proposed metabolic pathway for astilbin.

For quercitrin, a total of 11 metabolites (M1-M7, M11-M14) were identified, with M1-M7 being shared with astilbin. The observed biotransformations included the loss of Rha (M11), loss of Rha and 2H (M12), loss of Rha and O-methylenation (M13), and the loss of C_15_H_12_O_9_, oxidation, and C_6_H_8_O_6_ (M14). In the plasma samples taken 2 h after oral administration, quercitrin was found in low abundance, primarily in the form of its metabolites. The retention times of these metabolites ranged from 14.33 to 40.12 min, with the parent compound eluting at 36.46 min. Figure 8B illustrates the proposed metabolic pathway of quercitrin.

For engeletin, 13 metabolites (M15-M24) were identified. The biotransformations observed were the loss of Rha (M15), loss of Rha and O-methylenation (M16), loss of Rha and sulfate conjugation (M17), loss of Rha and glucuronidation (M18), loss of Rha, H_2_O, and O-methylenation (M19), loss of Rha and H_2_O (M20), loss of Rha, H_2_O, and glucuronidation (M21), loss of C_15_H_12_O_9_, oxidation, and C_6_H_8_O_6_ (M22), loss of C_15_H_12_O_9_, oxidation, and methylation (M23), and loss of C_15_H_12_O_9_ (M24). Engeletin was found in high abundance in plasma samples 2 h after oral administration. The retention times of its metabolites ranged from 15.61 to 40.12 min, with the parent compound eluting at 36.46 min. Figure 8C illustrates the proposed metabolic pathway of engeletin. The metabolic process in plasma involves reduction, oxidation, methylation, hydroxylation, glucuronidation, and sulfate conjugation.Thus, analyzing the differences in components at various time points provides insight into the dynamic changes of SGR *in vivo*. Additionally, the metabolic fingerprinting study of SGR suggests that the metabolites of different flavonoids exhibit similarities and form a complex metabolic network through intermediate metabolites.

### 3.6 Tissue distribution pharmacodynamic profiling study

#### 3.6.1 Characterization of absorbed flavonoids and metabolites in tissues

Currently, no studies have explored the tissue distribution of active components in rats following oral administration of SGR extracts. In this study, we investigated the distribution of SGR’s active components across nine tissues (heart, liver, spleen, lung, kidney, brain, stomach, small intestine, and large intestine) by optimizing UPLC-MS/MS separation conditions. The mass spectrometry parameters for the seven flavonoids in negative ion mode under optimal MRM conditions are listed in Supplementary Table S5. The UPLC liquid phase analysis conditions are shown in Supplementary Table S6. Chromatograms of the mixed flavonoid standards and drug-containing plasma samples are presented in Supplementary Figure S1, showing that the flavonoids were well separated. Considering the structural similarity among the flavonoids, the combined results of UPLC and MS/MS analyses enabled us to establish a quantification method for flavonoids. The calibration curves and concentration ranges for the seven flavonoids are listed in Supplementary Table S7, demonstrating that all seven flavonoids can be separated and quantified across a wide concentration range. The LOD and LOQ values for the flavonoids are also shown in Supplementary Table S7, with LOQ concentrations ranging between 0.5 and 1 ng/mL. Additionally, the distribution of 10 metabolites from SGR across the nine tissues was quantified. These metabolites were phase I and phase II products of the flavonoids astilbin, engeletin, and quercitrin, all sharing the same core structure as the parent compounds. The MS/MS parameters for the 10 metabolites are provided in Supplementary Table S8. Since no reference chemical standards were available, the concentrations of the metabolites were estimated semi-quantitatively using the parent compounds’ calibration curves. Supplementary Tables S9,10 present the distribution levels of the seven flavonoids and 10 metabolites across the nine tissues at 0.5 and 2 h post-administration.

**FIGURE 9:**
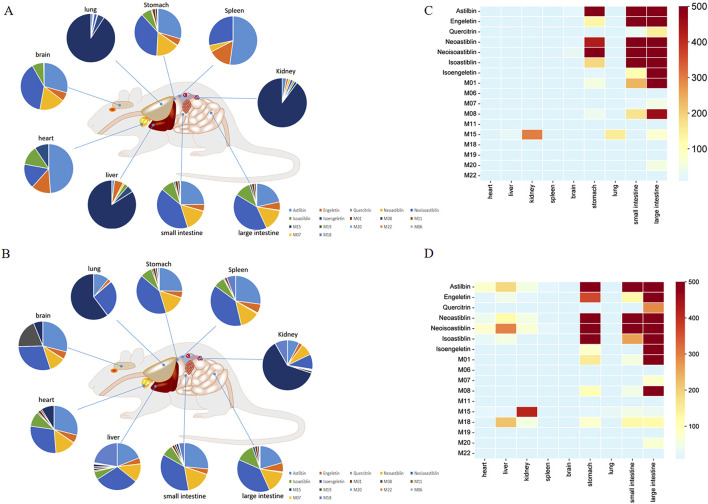
Exposure levels of 7 prototype compounds and 10 metabolites in plasma and tissues of SD rats at 0.5 **(A)** and 2 h **(B)** after the last oral administration of SGR. The percentage of exposure levels is displayed in pie charts. Prototype compounds are numbered as in Supplementary Table S8. Metabolite distribution in tissues at 0.5 **(C)** and 2 h **(D)** after the last oral administration of SGR. The color gradient from blue to red indicates increasing levels in the tissues.

As shown in Figures 9A,B, eight flavonoids and 10 metabolites were detected in plasma and tissues at 0.5 and 2 h post-administration. The results indicated that the large intestine, small intestine, stomach, liver, and kidney were the primary distribution organs for both the compounds and metabolites. These metabolites were primarily formed through glycoside loss, methylation, and glucuronidation of the flavonoid compounds. Figures 9C,D depict the tissue distribution of absorbed flavonoids and their metabolites at 0.5 and 2 h following oral administration of SGR. We found that in the sample extracted 0.5 h after administration, the metabolites were relatively small and mainly existed in the form of prototype compounds. 8 compounds were most distributed in the large intestine, and the top 3 compounds were Astilbin, Neoastiblin, and Neoisoastiblin. Ten metabolites were most distributed in the large intestine, including 9 phase I metabolites and 1 phase II metabolite. M01 (Loss Rha and H_2_O) from Astilbin and quercitrin ranked first in the large intestine, followed by M08 (Loss Rha) from Astilbin and M15 (Loss Rha) from Engeletin. In the small intestine and stomach, the largest distributions were M01, M08 and M20 (Loss Rha and H_2_O). M15 is most widely distributed in the liver and kidney. In the samples extracted 2 h after administration, metabolites of phase Ⅱ increased gradually, and both prototype compounds and metabolites existed simultaneously. 8 compounds were most distributed in the large intestine, and the top 3 compounds were Astilbin, Neoastiblin, and Neoisoastiblin. Twelve metabolites were most distributed in the large intestine, including 11 phase I metabolites and 1 phase II metabolite. The top 3 concentrations in the large intestine and stomach were M01, M08, and M18 (Loss Rha and Glucuronidation) from Engeletin. In the small intestine and liver, the top 3 were M01, M15, and M18. M08, M15, and M18 accounted for the top 3 in the kidney. Through the investigation of tissue distribution at various time points, we found that the prototype compound astilbin and its metabolites M1 and M8, along with the prototype compound engeletin and its metabolites M15 and M18, were the most widely distributed in the liver, intestines, kidneys, and stomach, which initially revealed the action relationship between the active ingredients in SGR and the target organs.

#### 3.6.2 Molecular docking analysis

**FIGURE 10:**
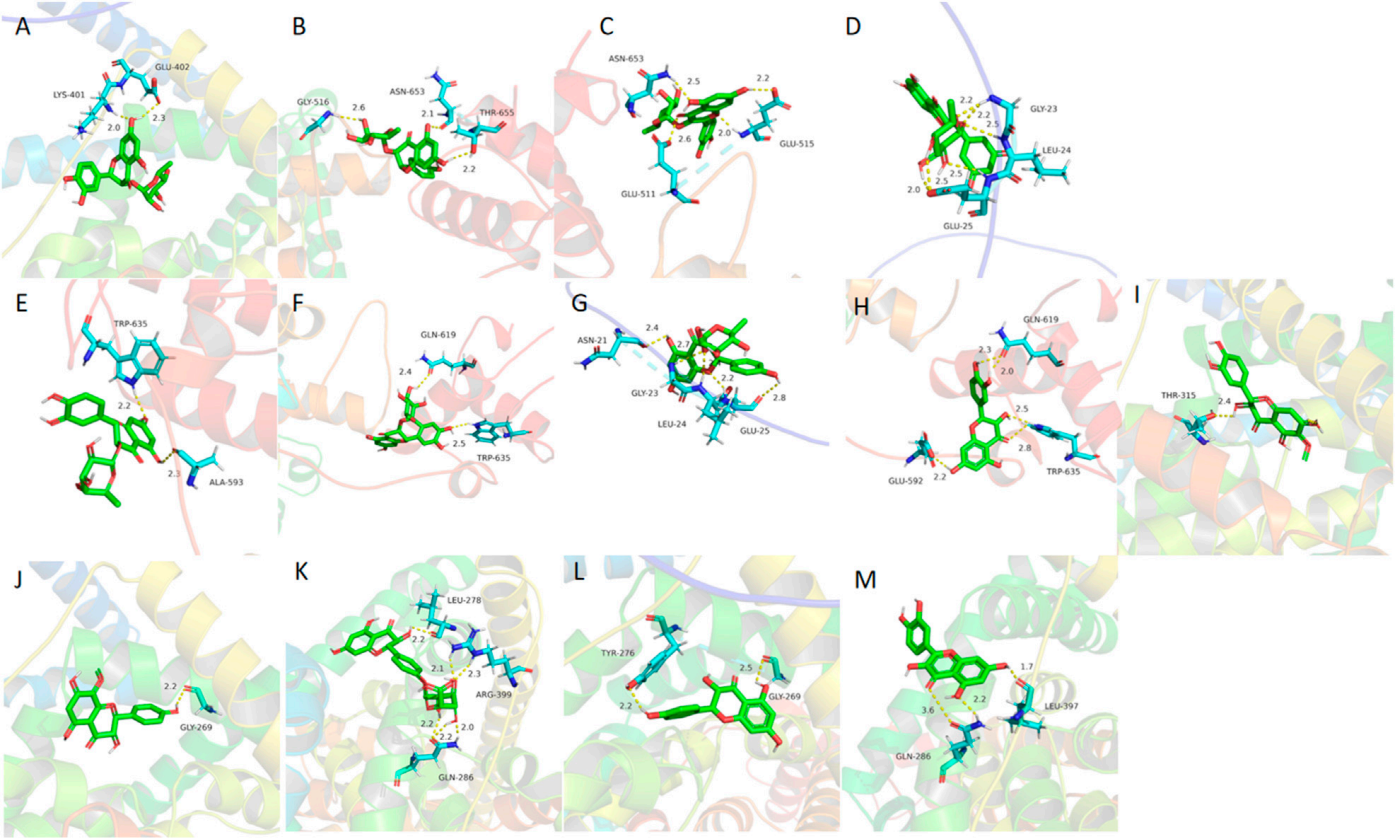
Molecular docking results of absorbed flavonoids from SGR **(A)** astilbin, **(B)** engeletin, **(C)** quercitrin, **(D)** isoastilbin, **(E)** neoastilbin, **(F)** neoisoastilbin, **(G)** isoengeletin and metabolites, **(H)** M08, **(I)** M09, **(J)** M16,**(K)** M18, **(L)** M15, **(M)** M11 in the active sites of the CNT2 homology models.

**FIGURE 11:**
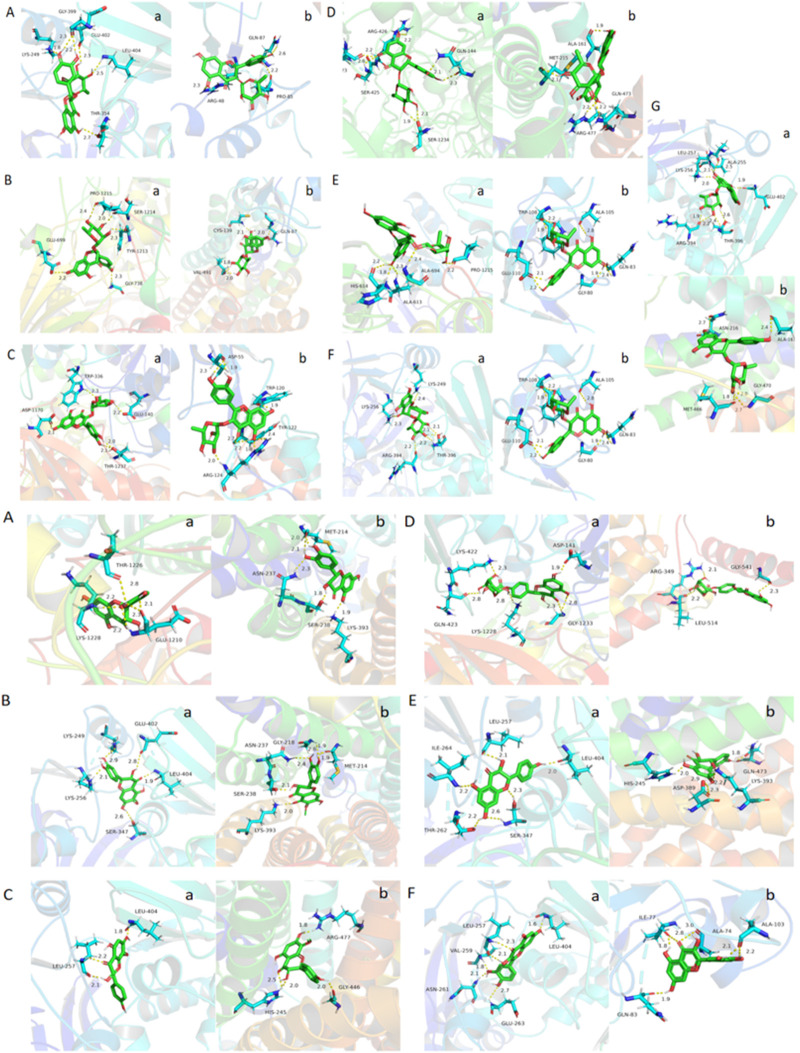
Molecular docking results of absorbed flavonoids from *SGR*: **(A)** astilbin, **(B)** engeletin, **(C)** quercitrin, **(D)** isoastilbin, **(E)** neoastilbin, **(F)** neoisoastilbin, **(G)** isoengeletin, and metabolites **(A)** M08, **(B)** M09, **(C)** M16, **(D)** M18, **(E)** M15, **(F)** M11 in the active sites of **(A)** XOD and **(B)** URAT1 homology models.

**FIGURE 12:**
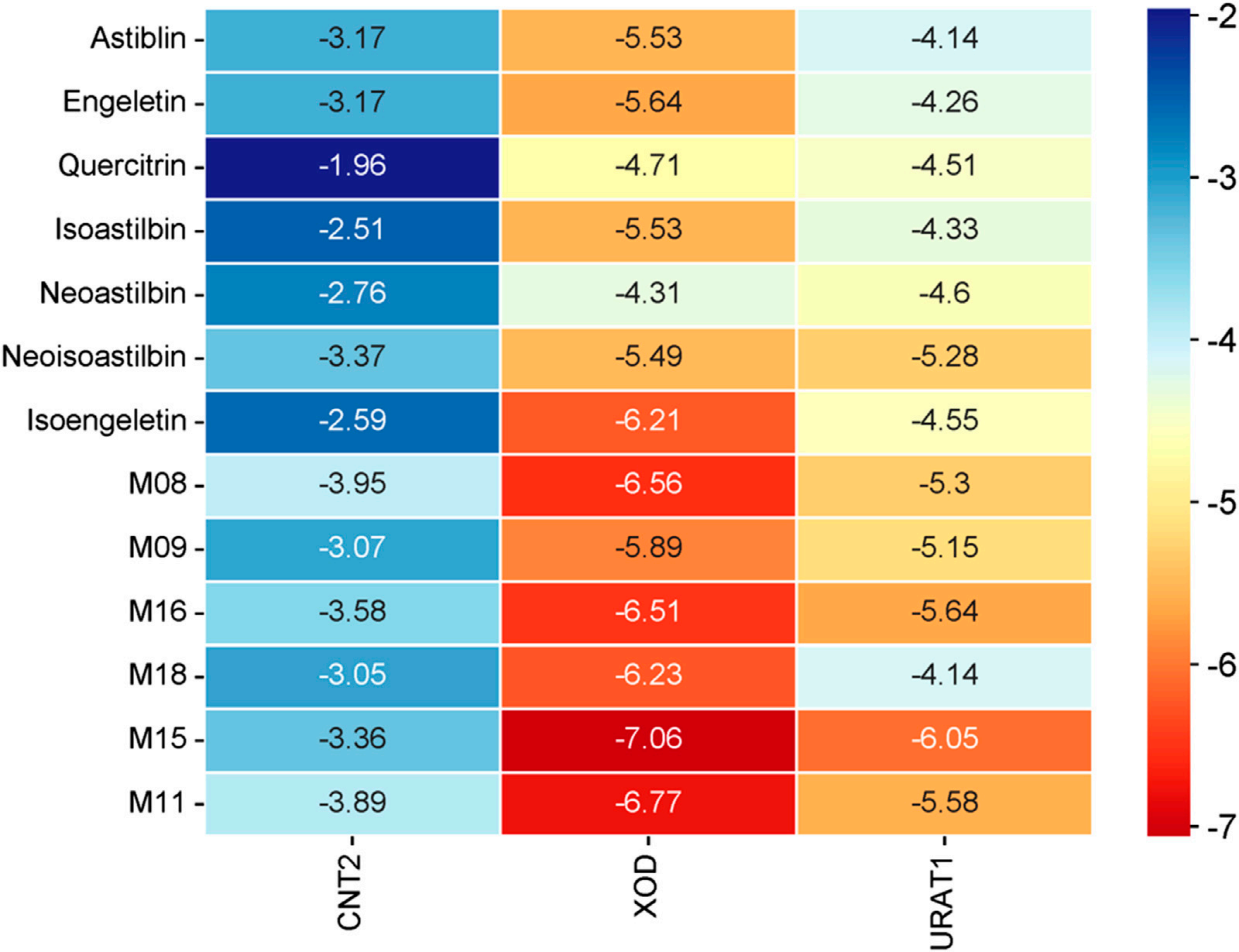
Heatmap of molecular docking score.

Molecular docking is a method used to predict the interactions between biological macromolecules and ligands. Previous network pharmacology studies on the treatment of hyperuricemia with SGR have mainly focused on anti-inflammatory-related target proteins (Shi et al., 2023), However, based on the theoretical framework of traditional Chinese medicine and relevant literature (Huang et al., 2019; Liang et al., 2019; Wang et al., 2019), the primary mechanism of SGR is to lower uric acid. Therefore, it is essential to select targets related to uric acid reduction for molecular docking analysis. The intestines play a crucial role in absorbing purine nucleosides from food. CNT2, encoded by the SLC28A2 gene, is the primary transporter for the intestinal absorption and transport of purine nucleosides (Tatani et al., 2016; Tatani et al., 2015). Inhibiting purine absorption in the intestines may therefore be a potential strategy for preventing and treating HUA. XOD, the rate-limiting enzyme in purine metabolism, is predominantly found in the liver of mammals. It catalyzes the oxidation of hypoxanthine to xanthine and subsequently the oxidation of xanthine to uric acid. Inhibiting XOD activity and blocking the formation of uric acid are key approaches in the treatment of hyperuricemia and gout (Alrashdi et al., 2022; Yong et al., 2022). Decreased uric acid excretion is a significant factor in the development of gout. The reabsorption and secretion of urate in the kidneys are mediated by various urate transporters, with URAT1 playing a major role. URAT1 is responsible for approximately 90% of uric acid reabsorption in the kidneys and is a critical target for hyperuricemia treatment (Chen et al., 2021; Li et al., 2021). Based on these considerations, we selected CNT2 (which affects the transport of uric acid precursors in the gut), XOD (which influences uric acid production in the liver), and URAT1 (a protein that affects uric acid transport and reabsorption in the kidneys) as the target proteins for molecular docking. Figures 10, 11 illustrate the detailed interaction patterns of the seven compounds and six metabolites with the target proteins CNT2, XOD, and URAT1, while Figure 12 shows the docking binding energies. The docking results were supported by the distribution of these compounds in the intestine, liver, and kidney. M8 exhibited the lowest docking binding energy (−3.95) with CNT2, indicating the strongest binding activity. This suggests that M8 may reduce the formation of uric acid precursors by inhibiting CNT2 activity. For XOD, the docking binding energies of five compounds (astilbin, isoastilbin, neoisoastilbin, engeletin, and isoengeletin) and six metabolites (M08, M09, M11, M15, M16, and M18) were less than −5. The strongest binding activities were observed with M15 (−7.06) and M11 (−6.77), followed by M08 (−6.56). These metabolites may inhibit uric acid production by blocking XOD activity. In terms of URAT1, neoisoastilbin (−5.28) and six metabolites (M08, M09, M11, M15, M16, and M18) showed docking binding energies below −5. The strongest binding energies were recorded for M15 (−6.05) and M16 (−5.64), followed by M11 (−5.58) and M08 (−5.30). These compounds may inhibit uric acid reabsorption in renal tubules by affecting the URAT1 transport protein. Molecular docking identified compounds and metabolites with strong correlations to CNT2, XOD, and URAT1, further validating the relationship between plasma metabolic profiles and tissue-distributed pharmacodynamic profiles.

## 4 Discussion

Fingerprinting of traditional Chinese medicine is the application of modern science and technology to obtain the structural information of components or characteristic parts of traditional Chinese medicine under the guidance of the holistic view of traditional Chinese medicine, which has the advantages of systematicity, characterization, and good reproducibility (Du et al., 2024a). The combination of fingerprinting and pharmacodynamic effects enables a more comprehensive and integrated evaluation of the quality and safety of traditional Chinese medicines and a more efficient identification of the pharmacodynamic substances and mechanisms of action of traditional Chinese medicines (Tang et al., 2024). To our knowledge, current studies on the uric acid-lowering pharmacodynamics of SGR include the anti-inflammatory effects of the ethanol crude extract of SGR (S. Wang et al., 2019), and the uric acid-lowering effects of the monomer components in SGR on hyperuricemic mice (Huang et al., 2019), but these studies still have the limitation of unclear pharmacodynamic substances. As we know, after oral absorption, drugs will be metabolized and transformed *in vivo* to exert their medicinal effects, and a comprehensive study of chemical, metabolic, and medicinal components is an effective means to clarify the pharmacodynamic substances of SGR for the treatment of hyperuricemia. On the other hand, the extraction method of standard decoction is not only green and environmentally friendly but also can provide a basis for the consistency of clinical dosage. Therefore, the present study was guided by Chinese medicine theory, combined with pharmacodynamic experiments, and for the first time, chemical fingerprinting, metabolic fingerprinting, and pharmacodynamic fingerprinting were combined to systematically evaluate the material basis and mechanism of action of the standard decoction of SGR. From the perspective of lowering serum uric acid level, inhibiting XOD activity, and promoting uric acid excretion, we initially revealed the pathway of uric acid-lowering action of the medium-polar compound group in SGR, and the large-polar compound group, mainly containing phenolic acids, had a non-significant effect. The results of the experiments also showed that astilbin, one of the quality markers in SGR, has been reported to have good effects in uric acid reduction, renal protection, and antioxidant (Yang and Zhang, 2022), and we found that its isomer, neoisoastiblin, and metabolite M08, which not only have high levels in plasma and tissues but also bind stably to three uric acid-lowering-related target proteins, may be a core component in uric acid lowering. However, no relevant studies have been reported, so this study can provide a reference for the new development and design of SGR flavonoids in the future.

In the pharmacodynamic study, the ingredients in the SGR standard decoction were firstly divided into two groups by using the macroporous resin separation method, and the UPLC-Q-TOF/MS technique was used to identify the ingredients in each group. The group of large-polar compounds contained six organic acids and three flavonoids, and the group of medium-polar compounds contained 12 flavonoids, and the cleavage patterns of six common flavonoids were analyzed. We found that flavonoids in SGR have similar cleavage pathways, including glycosidic bond breaking and phenolic hydroxyl oxidation, and that analysis of the cleavage patterns of flavonoids can provide predictions for *in vivo* metabolic pathways. Next, we compared the uric acid-lowering effects of the various polar chemical groups of SGR. A HUA mouse model was established by intraperitoneal injection of potassium oxonate, and serum uric acid levels, renal uric acid levels, and hepatic xanthine oxidase (XOD) activity were compared between the control group, the model group, and the groups administered different polarities of the SGR extract. This allowed us to identify the effective uric acid-lowering components. The medium-polar compound group was found to be the effective uric acid-lowering component of SGR, which included neoastilbin, astilbin, isoastilbin, neoisoastilbin, engeletin, quercitrin, isoengeletin, and other flavonoids.

We have preliminarily clarified the pharmacologically active components of SGR through mouse pharmacodynamic experiments, but the pathways of action for lowering uric acid may be multiple, such as inhibiting uric acid production through inhibition of XOD enzyme activity, and affecting the URAT1 uric acid transporter to promote uric acid excretion etc., (Du et al., 2024b), but there is still no relevant study that elucidates the relationship between the pharmacodynamic substances and the metabolic pathways of SGR. Therefore, we further analyzed the *in vivo* absorption and metabolic composition of flavonoids in SGR. A total of eight parent compounds and 24 metabolites were identified in rat plasma and urine samples, primarily resulting from redox reactions, hydroxylation, methylation, glucuronidation, and sulfate conjugation of flavonoid glycosides after the loss of glycosides. Evaluating the efficacy of SGR through phase I/II metabolites may provide better insights, as the parent compounds undergo metabolism. For example, metabolites such as M1 (loss of Rha and H_2_O, RT = 31.86 min, observed in both astilbin and quercitrin metabolic profiles), M17 (loss of Rha and sulfate conjugation, RT = 23.29 min, detected in the engeletin pathway), and M18 (loss of Rha and glucuronidation, RT = 35.83 min, also found in the engeletin pathway) were identified. We discovered that these three flavonoids share similar metabolic pathways, which intersect through common intermediate metabolites.It has been found that hydrogen bonding and hydrophobic interaction forces are easily formed between flavonoids and XOD or URAT1, probably because most flavonoids contain multiple hydroxyl groups, which can easily bind to polar amino acid residues of XOD or URAT1 through hydrogen bonding (Guo et al., 2025; Xue et al., 2023). Besides, flavonoids can reduce the activity of XOD through competitive inhibition, but when the molecular weight of flavonoids is too large, the repulsive interactions between them and the amino acid binding sites in XOD may cause a spatial site-blocking effect, which reduces the inhibitory activity of flavonoids on XOD (Cheng-yuan & Jian-gang, 2023), whereas the main metabolites of flavonoids *in vivo* in our study were produced by the loss of one molecule of glycoside, therefore, the compounds absorbed in SGR may increase the inhibitory activity of XOD due to the loss of glycosidic bonds, which also suggests that summarizing the metabolic pathways of the active components in SGR from a structure-activity perspective is important for grasping the mechanisms related to the treatment of HUA with SGR.

In the tissue distribution study, flavonoids and metabolites from SGR were examined across nine tissues. The results indicated that three metabolites M15, M01, and M08 were more widely distributed than others, with a higher concentration in the liver, kidneys, small intestine, large intestine, and stomach. These metabolites likely play a significant role in the *in vivo* activity of SGR extracts. Specifically, M08 and M15 are metabolites of astilbin and engeletin following glycoside loss, while M01 is a metabolite of both astilbin and quercitrin following the loss of glycosides and H_2_O. Therefore, SGR extract may exert its effect through a combination of flavonoids and their metabolites, with the liver, large intestine, small intestine, and kidneys being the primary target organs. Additionally, hyperuricemia has been associated with heart failure (Si et al., 2021), diabetes (Johnson et al., 2013), and stroke (Weir et al., 2003). Consequently, we also analyzed the distribution of SGR’s active components in the heart, spleen, lungs, brain, and stomach, providing a reference for the potential management of other disorders.

Xanthine oxidase (XOD) in the liver catalyzes the oxidation of hypoxanthine (HX) to xanthine and subsequently to UA, making XOD the rate-limiting enzyme in purine metabolism (Sun et al., 2021). Therefore, inhibiting XOD activity is a primary therapeutic target for reducing UA synthesis (Kohagura et al., 2016). Numerous epidemiological studies have shown that excessive intake of purine-rich foods is a significant risk factor for hyperuricemia. Since CNT2 is the primary transporter for purine nucleosides during intestinal absorption and transport, inhibiting CNT2 can reduce gastrointestinal absorption of purines, thereby helping to prevent and manage hyperuricemia (Hiratochi et al., 2012). The kidneys are the primary organs responsible for uric acid excretion, accounting for roughly two-thirds of the total excretion. URAT1, a uric acid reabsorption protein, is responsible for about 90% of uric acid reabsorption in the kidneys, making it a key therapeutic target for hyperuricemia treatment (Yusnaini et al., 2023). To identify the key active components of SGR that regulate UA metabolism along the liver-intestine-kidney axis, we performed molecular docking of seven flavonoids and six metabolites from SGR with the target proteins CNT2, XOD, and URAT1, which correspond to these organs. The docking results revealed that six metabolites (M08, M09, M16, M18, M15, and M11) had better binding scores with the three target proteins than the five parent compounds (astilbin, engeletin, isoastilbin, neoisoastilbin, and isoengeletin). We analyzed protein-ligand interactions, including hydrophobic, hydrogen-bonding, water-bridging, and ionic interactions, in all compounds and metabolites in SGR (Du et al., 2024c; Zheng et al., 2024). Among the metabolites, M15, M16, M08, and M11 had the strongest binding activity to target proteins. M08 had the highest binding activity to CNT2, which mainly binds to CNT2-associated residues (including GLU592, TRP635, and GLN619) and reduces the synthesis of uric acid precursors by interfering with CNT2. M15 and M11 have the strongest binding affinity for XOD, and they inhibit the catalytic activity of XOD by binding to residues in the XOD active site pocket (including LEU257, LEU404, SER347, VAL259, and ASN261) mainly through hydrophobic interactions, thus reducing the formation of UA. M15 and M16 have the best binding activity to URAT1, and they inhibit the transport activity of URAT1 by targeting residues in the URAT1 active site pocket (including HIS245, ASP389, GLN473, ARG477, and GLY446), thereby reducing uric acid reabsorption. Thus, the flavonoids and their metabolites in SGR may affect their binding modes and sites with CNT2, XOD, and URAT1 through competitive inhibition, and affecting their activities. These findings fully reflect the multicomponent, multi-target mechanism by which SGR reduces uric acid levels. On the other hand, the compounds and metabolites in SGR showed more stable binding to XOD and URAT1 than the target protein CNT2, and it suggest that SGR may be a potential candidate for drug design or functional food for the treatment of hyperuricemia, and based on the clarification of the conformational relationship of SGR, the enhancement of the inhibitory activity of XOD and URAT1 through structural modification may be an important future research direction.

To the best of our understanding, this is the first systematic and comprehensive assessment of the efficacy and material basis of SGR standard decoction for the treatment of HUA. However, this study has some limitations. The studies on the core target proteins and key signaling pathways for uric acid lowering are incomplete, and the studies on drug toxicity are insufficient. Therefore, we will next explore the mechanism of action and drug safety of SGR standard soup for uric acid lowering by more pharmacological experimental studies.

## 5 Conclusion

This study presents an efficient and rapid method for comprehensively characterizing the active ingredients in *Smilax glabra* Roxb standard decoction. By utilizing UPLC-Q-TOF/MS to generate chemical fingerprints of SGR and validating them through pharmacodynamic experiments, the study quickly identified the uric acid-lowering active ingredient groups in SGR. The method is environmentally friendly and offers good reproducibility. Through the combination of metabolic fingerprinting, tissue distribution studies, and molecular docking, the active ingredients responsible for lowering uric acid were identified. Eight absorbed flavonoids and 24 metabolites were detected in the metabolic fingerprinting analysis. The pharmacodynamic fingerprinting revealed that the main distribution sites for these flavonoids and their metabolites were the liver, intestines, kidneys, and stomach, with M15, M01, and M08 being the most widely distributed metabolites. Molecular docking further validated that the metabolites with the strongest binding affinity to the target proteins CNT2, XOD, and URAT1 were M15, M16, M08, and M11, respectively. This study offers a new approach for analyzing the pharmacodynamic mechanisms of SGR and guiding its clinical use, as well as providing a methodological reference for the rapid screening of pharmacodynamically active ingredients in other herbal medicines.

## Data Availability

The original contributions presented in the study are included in the article/Supplementary Material, further inquiries can be directed to the corresponding authors.
