# Supplementary material for: Comprehensive characterisation of the active ingredients of Smilax glabra Roxb based on chemical fingerprinting, metabolic fingerprinting and pharmacodynamic fingerprinting
Source: Front Pharmacol. 2025 Apr 23;16:1519054. doi: 10.3389/fphar.2025.1519054 (PMC12055767; doi:10.3389/fphar.2025.1519054)
Supplement: Supplementary file 1 [file DataSheet1.docx]

Supplementary Material

# Supplementary Figures and Tables

## Supplementary Figures


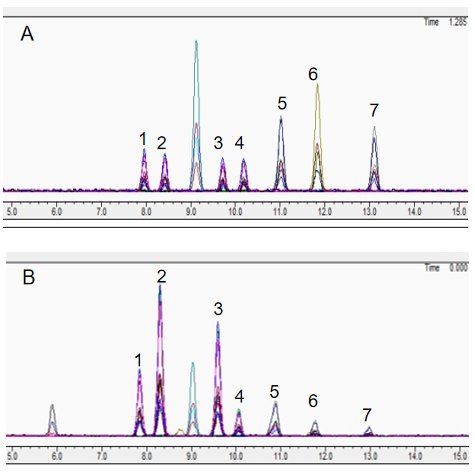


**Supplementary Figure 1.** The chromatograms of the mixed standards of the 7 flavonoids and the chromatograms of the drug-containing plasma samples.

## Supplementary Tables

**Supplementary Table 1.** Establishment of hyperuricaemia model in mice

| Time（h） | Sample Absorbance Value | Standard absorbance value | Serum uric acid (UA) level（µmol/L） |
| --- | --- | --- | --- |
| 0 | 0.0811±0.0048 | 0.0859 | 280.6±14.87 |
| 0.5 | 0.0867±0.0026 | 0.0859 | 300.0±12.40 |
| 1 | 0.0922±0.0039 | 0.0859 | 319.4±14.28** |
| 1.5 | 0.0864±0.0041 | 0.0859 | 299.1±14.02 |
| 2 | 0.0821±0.0038 | 0.0859 | 284.4±14.87 |

**P<0.01 Highly significant difference compared to no injection of potassium oxonate.

**Supplementary Table 2.** Effect on serum uric acid in hyperuricaemia model mice

| Groups | Dose | Serum uric acid (UA) level（µmol/L） |
| --- | --- | --- |
| NC | / | 282±12.38 |
| HUA | / | 319.06±21.01** |
| AP | 10 mg/kg | 255.3±14.99^##^ |
| LPC | 100 mg/kg | 332.3±12.96 |
| MPC | 100 mg/kg | 283.5±23.99^##^ |

(NC: normal control; HUA: hyperuricemia; AP: allopurinol; BZM: benz-bromarone; LPC: Large polar compound group; MPC: Medium polar compound group. **p< 0.01 vs. NC group; ^##^p< 0.01 vs. HUA group, n = 6 for each group.

**Supplementary Table 3.** Effect on xanthine oxidase (XOD) activity in mouse liver

| groups | dose | Serum uric acid value（µmol/L） |
| --- | --- | --- |
| NC | / | 18.43±1.28 |
| HUA | / | 18.09±1.80 |
| AP | 10 mg/kg | 13.77±1.12^#^^#^ |
| LPC | 100 mg/kg | 19.03±2.30 |
| MPC | 100 mg/kg | 15.28±1.19^#^ |

(NC: normal control; HUA: hyperuricemia; AP: allopurinol; BZM: benz-bromarone; LPC: Large polar compound group; MPC: Medium polar compound group. ^#^^#^p< 0.01 vs. HUA group; ^#^p < 0.05 vs. HUA group, n = 6 for each group.

**Supplementary Table 4.** Effect on renal uric acid in mice

| groups | dose | Serum uric acid value（µmol/L） |
| --- | --- | --- |
| NC | / | 179.9±22.75 |
| HUA | / | 182.7±26.39 |
| AP | 10 mg/kg | 78.75±7.02ΔΔ |
| LPC | 100 mg/kg | 182.7±22.31 |
| MPC | 100 mg/kg | 118.4±22.36Δ |

(NC: normal control; HUA: hyperuricemia; AP: allopurinol; BZM: benz-bromarone; LPC:Large polar compound group; MPC:Medium polar compound group. ^##^p< 0.01 vs. HUA group; ^#^p < 0.05 vs. HUA group, n = 6 for each group.

**Supplementary Table 5.** The optimized MS analytical parameters of 7 flavonoids of SGR

| compounds | retention time (min) | Precursor (m/z) | Product ion (m/z) | Q1,V | CE,V | Q3,V |
| --- | --- | --- | --- | --- | --- | --- |
| neoastiblin | 8.18 | 449.2 | 151.05、285.10、303.15 | 15 | 22 | 14 |
| astilbin | 8.66 | 449.25 | 151.05、285.15、303.10 | 11 | 24 | 12 |
| neoisoastiblin | 9.98 | 449.25 | 151.05、285.10、303.25 | 30 | 23 | 14 |
| isoastiblin | 10.47 | 449.2 | 151.05、285.10、303.05 | 22 | 22 | 12 |
| engeletin | 11.28 | 433.2 | 152.00、269.15、180.05 | 29 | 35 | 29 |
| quercitrin | 12.12 | 447.15 | 300.10、301.05、271.05 | 16 | 27 | 30 |
| isoengeletin | 13.32 | 433.2 | 151.95、269.15、180.10 | 11 | 37 | 26 |

**Supplementary Table 6.** UPLC liquid chromatography gradient elution procedure

| Time(min) | Solution A(%) | Solution B(%) |
| --- | --- | --- |
| 0 | 70 | 30 |
| 1 | 70 | 30 |
| 14 | 50 | 50 |
| 14.1 | 70 | 30 |
| 15 | 70 | 30 |

**Supplementary Table 7.** Calibration curve, correlation coefficient, LOD and LOQ of 7 flavonoids of SGR

| Analytes | Standard curves | Calibration range(ng/mL) | R^2^ | LOD（ng/mL) | LOQ（ng/mL) |
| --- | --- | --- | --- | --- | --- |
| neoastiblin | y = 518.51x + 8048.1 | 1.5-500 | 0.998 | 0.2 | 1 |
| astilbin | y = 597.04x + 15758 | 1-500 | 0.999 | 0.2 | 1 |
| neoisoastiblin | y = 253.47x + 10021 | 1-500 | 0.991 | 0.2 | 1 |
| isoastiblin | y = 401.37x + 1243 | 1-500 | 0.996 | 0.2 | 1 |
| engeletin | y = 699.41x + 4108.3 | 0.5-500 | 0.998 | 0.1 | 0.5 |
| quercitrin | y =1943.9x + 6875.2 | 0.5-500 | 0.999 | 0.1 | 0.5 |
| isoengeletin | y =699.41x +4108.3 | 0.5-500 | 0.999 | 0.1 | 0.5 |

**Supplementary Table 8.** The optimized MS analytical parameters of 10 metabolites of SGR

| Met | [M−H]^−^(m/z) | MS/MS fragments | Parent |
| --- | --- | --- | --- |
| M01 | 285.0404 | 259,179,151 | astilbin |
| M06 | 155.035 | 151,137,107 | astilbin |
| M07 | 125.0244 | 116,112,107 | astilbin |
| M08 | 303.051 | 285,151,125 | astilbin |
| M11 | 301.0353 | 287,248,175 | quercitrin |
| M15 | 287.0561 | 269,151,107 | engeletin |
| M18 | 463.0882 | 433,317,299 | engeletin |
| M19 | 299.0561 | 269,179,116 | engeletin |
| M20 | 269.0455 | 175,151,107 | engeletin |
| M22 | 317.0656 | 299,269,178 | engeletin |

**Supplementary Table 9.** Tissue Distributions of 8 compounds and 7 metabolites 2h after the oral administration of SGR

| Tissue | Astilbin（ng/  mL) | Engeletin（ng/  mL) | Quercitrin（ng/  mL) | Neoastiblin（ng/  mL) | Neoisoastiblin（ng/  mL) | Isoastiblin（ng/  mL) | Isoengeletin（ng/  mL) | M01（ng/mL) | M06（ng/mL) | M07（ng/mL) | M08（ng/mL) | M11 （ng/mL) | M15（ng/  mL) | M18（ng/  mL) | M19（ng/mL) | M20（ng/mL) | M22（ng/mL) |
| --- | --- | --- | --- | --- | --- | --- | --- | --- | --- | --- | --- | --- | --- | --- | --- | --- | --- |
| heart | 79.7±8.5 | 15.2±2.4 | 0.8±0.2 | 38.8±7.2 | 78.6±18 | 28±5.5 | 2.3±0.4 | 5.3±0.8 | ND | ND | 3±0.9 | ND | 24.2±10.1 | ND | ND | ND | ND |
| liver | 185.6±10.2 | 40.9±12.6 | 1.5±0.4 | 123.5±12.5 | 296.5±21.2 | 54±12.5 | 8.2±1.5 | 13.8±1.8 | 3.7±0.9 | 1.2±0.4 | 11.7±2.2 | ND | 18.4±3.8 | 218.4±35 | 2.5±1.2 | ND | 2.8±1.6 |
| kidney | 51.8±4.8 | 16.1±4.1 | 0.4±0.2 | 52.2±8.6 | 65.8±12.2 | ND | 4.2±0.8 | 2.9±0.4 | 0.4±0.1 | ND | 9.5±3.5 | ND | 416.9±18.5 | 55.3±21.2 | ND | ND | ND |
| spleen | 17.5±4.2 | 3.8±2 | 0.4±0.3 | 8.2±2.4 | 25±4.8 | 4.4±2.8 | 0.4±0.1 | 1±0.2 | ND | ND | ND | ND | ND | 3.7±1.2 | ND | ND | ND |
| brain | 4.2±1.5 | 0.7±0.3 | 0.1±0.2 | 1.5±0.5 | 4.3±2.2 | ND | ND | ND | ND | ND | 2.8±0.6 | ND | 0.9±0.2 | ND | ND | ND | ND |
| stomach | 2048.1±25.5 | 362.5±35 | 18.4±2.5 | 1231.1±36 | 3305.3±25.5 | 666.1±26.1 | 80.4±18 | 154.7±20.2 | 5±1.8 | 12.4±4.6 | 104.6±24.4 | 0.2±0.1 | 18.4±9.6 | 76.7±16 | 1.3±0.4 | 6.8±1.2 | 0.5±0.2 |
| lung | 5.7±2.4 | 1.1±0.2 | 0.2±0.2 | ND | 13.3±2.4 | ND | ND | ND | ND | ND | ND | ND | 30.8±9.2 | ND | ND | ND | ND |
| small intestine | 892.5±28.8 | 126.1±16.4 | 7.1±2.5 | 567.7±35.3 | 1214.9±32 | 259.5±36.2 | 12.6±2 | 57.5±12 | 3.2±1.8 | 5.5±1.5 | 46.1±26.0 | ND | 49.8±12.5 | 125.5±18.5 | 1.4±0.5 | 2.5±0.5 | 1.6±0.8 |
| large intestine | 9949.1±35.2 | 3117.5±25 | 285±28 | 7579.3±32 | 18557.3±48.5 | 6280.9±55.9 | 801.4±20.5 | 875.9±26.4 | 4.6±2 | 76.7±28.8 | 627.2±66.2 | ND | 35.9±8.5 | 124.7±16.6 | 1.4±1 | 72.9±12.8 | 1.9±1.5 |

**Supplementary Table 10.** Tissue Distributions of 8 compounds and 7 metabolites 0.5h after the oral administration of SGR

| Tissue | Astilbin（ng/  mL) | Engeletin（ng/  mL) | Quercitrin（ng/  mL) | Neoastiblin（ng/  mL) | Neoisoastiblin（ng/  mL) | Isoastiblin（ng/  mL) | Isoengeletin（ng/  mL) | M01（ng/  mL) | M06（ng/mL) | M07（ng/mL) | M08（ng/mL) | M11（ng/mL) | M15（ng/mL) | M18（ng/mL) | M19（ng/mL) | M20（ng/mL) | M22（ng/mL) |
| --- | --- | --- | --- | --- | --- | --- | --- | --- | --- | --- | --- | --- | --- | --- | --- | --- | --- |
| heart | 4.2±1.2 | 1.1±0.3 | ND | ND | 1.4±0.4 | 1.1±0.4 | 0.8±0.2 | ND | ND | ND | ND | ND | ND | ND | ND | ND | ND |
| liver | 0.6±0.3 | 2±0.4 | ND | 0.4±0.1 | ND | 0.9±0.3 | 1.6±0.2 | ND | ND | ND | ND | ND | 29.1±5.5 | ND | ND | ND | ND |
| kidney | 9.2±2.2 | 4.8±1.2 | ND | 4.7±0.4 | 5.7±1.2 | 4±1.2 | 5.7±2.4 | ND | ND | ND | ND | ND | 299.4±38.8 | ND | ND | ND | ND |
| spleen | 2.4±0.3 | 0.7±0.2 | ND | 0.2±0.1 | 1.3±0.4 | ND | ND | ND | ND | ND | ND | ND | ND | ND | ND | ND | ND |
| brain | 23.4±3.2 | 4.8±1.5 | ND | 14±2.8 | 30.9±1.8 | 6.3±1.8 | 0.3±0.1 | ND | ND | ND | ND | ND | ND | ND | ND | ND | ND |
| stomach | 786.8±21 | 130.1±18.8 | 10.1±2.5 | 429.4±28 | 996.5±27.5 | 186.6±18.6 | 19.5±3.6 | 56.4±18.2 | 0.9±0.3 | 4.9±1.3 | 40.4±12.5 | 0.6±0.3 | 2±0.5 | ND | ND | 3.3±0.6 | ND |
| lung | 2.4±0.3 | 0.6±0.2 | ND | 0.7±0.2 | 3.3±0.6 | 0.8±0.5 | 7.6±1.6 | ND | ND | ND | ND | ND | 152.5±25.2 | ND | ND | ND | ND |
| small intestine | 2974.5±36 | 540.7±62.2 | 33.4±11.3 | 1866.3±41.3 | 4910±48 | 1085.7±55.6 | 118.5±21.5 | 237.4±24 | 8.7±2.5 | 21.3±6.6 | 168.1±22.3 | 0.2±0.2 | 8.6±2.5 | ND | 4.2±2.5 | 12.2±2.4 | 2.1±0.5 |
| large intestine | 7746.7±55.6 | 2063.6±53.2 | 146.2±23.2 | 5516.1±42.5 | 14568.4±122.2 | 4072±82 | 506.1±52.6 | 646.1±36.5 | 2±0.6 | 48.8±12.5 | 461.3±32 | ND | 74.2±14.5 | ND | ND | 51.1±18 | ND |
